# Advancements, measurement uncertainties, and recent comparisons of the NOAA frost point hygrometer

**DOI:** 10.5194/amt-9-4295-2016

**Published:** 2016-09-05

**Authors:** Emrys G. Hall, Allen F. Jordan, Dale F. Hurst, Samuel J. Oltmans, Holger Vömel, Benjamin Kühnreich, Volker Ebert

**Affiliations:** 1 Cooperative Institute for Research in Environmental Sciences, University of Colorado, Boulder, Colorado, USA; 2 NOAA Earth System Research Laboratory, Global Monitoring Division, Boulder, Colorado, USA; 3 National Center for Atmospheric Research, Earth Observation Laboratory, Boulder, Colorado, USA; 4 Physikalisch-Technische Bundesanstalt, Braunschweig, Germany; 5 Center of Smart Interfaces, Technische Universität Darmstadt, Darmstadt, Germany

## Abstract

The NOAA frost point hygrometer (FPH) is a balloon-borne instrument flown monthly at three sites to measure water vapor profiles up to 28 km. The FPH record from Boulder, Colorado, is the longest continuous stratospheric water vapor record. The instrument has an uncertainty in the stratosphere that is < 6 % and up to 12 % in the troposphere. A digital microcontroller version of the instrument improved upon the older versions in 2008 with sunlight filtering, better frost control, and resistance to radio frequency interference (RFI). A new thermistor calibration technique was implemented in 2014, decreasing the uncertainty in the thermistor calibration fit to less than 0.01 °C over the full range of frost – or dew point temperatures (−93 to +20 °C) measured during a profile. Results from multiple water vapor intercomparisons are presented, including the excellent agreement between the NOAA FPH and the direct tunable diode laser absorption spectrometer (dTDLAS) MC-PicT-1.4 during AquaVIT-2 chamber experiments over 6 days that provides confidence in the accuracy of the FPH measurements. Dual instrument flights with two FPHs or an FPH and a cryogenic frost point hygrometer (CFH) also show good agreement when launched on the same balloon. The results from these comparisons demonstrate the high level of accuracy of the NOAA FPH.

## 1 Introduction

Water vapor is the most abundant and important greenhouse gas in the atmosphere and contributes to many processes and feedback mechanisms (Dessler et al., 2008). Though the vast majority of this highly variable invisible gas is found near the surface, water vapor in the upper troposphere and stratosphere can significantly influence climate (Held and Soden, 2000).

The concentration of water vapor in the stratosphere is controlled by transport through the tropical tropopause layer (TTL) rendering the stratospheric air extremely dry through a freeze-drying process (Brewer, 1949). The seasonal cycle of the tropical tropopause temperature controls the seasonal cycle of water vapor entering the stratosphere which is advected upward and poleward (Mote et al., 1996). Methane oxidation is a photochemical source of water vapor in the atmosphere but can only account for 25–34 % of the net stratospheric water vapor increase between 1978 and 2003 (Rohs et al., 2006). Water vapor plays a role in cirrus cloud formation, which impacts Earth’s radiation budget. High thin cirrus clouds may cool the surface while producing a net warming in the stratosphere by absorbing outgoing longwave radiation in the atmosphere (Lee et al., 2009).

The Boulder stratospheric water vapor record shows a net increase of ~ 1 ppmv (27 %) since 1980 (Hurst et al., 2011a) with a sharp decrease of roughly 10 % in 2001 to 2005 (Randel et al., 2006; Rosenlof and Reid, 2008). Solomon et al. (2010) estimated that this sharp drop in stratospheric water vapor in 2001 decelerated the global surface temperature increase by approximately 25 % compared to what would have been caused by well-mixed greenhouse gases between 2000 and 2009. It is imperative to monitor the distribution of water vapor, especially in the stratosphere where it represents an important driver of decadal worldwide surface climate change (Solomon et al., 2010).

Accurate measurements of upper tropospheric and lower stratospheric (UTLS) water vapor are pivotal for understanding changes in stratospheric water vapor and their impact on the radiative forcing (Forster and Shine, 2002) of our climate. Müller et al. (2016) argue for a large network of frost point hygrometers (FPHs) with global coverage spanning many decades because of the climatic importance of water vapor. Worldwide there are more than 800 radiosonde stations launching balloon instruments twice daily, totaling > 500 000 vertical profiles of wind speed/direction, pressure, temperature, and humidity each year. These measurements are predominately used for weather forecasting. While radiosonde wind, pressure, and temperature measurements are accurate up to balloon burst (> 30 km), the relative humidity measurements often lack the precision and accuracy necessary for climate research and are most useful in the low to mid-troposphere. Stratospheric water vapor is deceptively hard to measure, despite the relatively high concentration (> 1 ppmv) compared to most trace gases in the atmosphere. Vertical profiles of UTLS water vapor have been measured by satellite, aircraft, lidar, and balloon-borne instruments, revealing considerable disagreements (~ 25–100 %) between a core group of stratospheric water vapor measurements (Kley et al., 2000; Weinstock et al., 2009). A 2011 intercomparison of two frost point hygrometers and five in situ aircraft hygrometers show reduced but still statistically significant differences of 0.4–0.8 ppmv (10–20 %) (Rollins et al., 2014). Corroborating these findings, Meyer et al. (2015) show that agreement between a core group of hygrometers has improved over the past 2 decades from ±30 % or more to approximately ±5–20 % for mixing ratios under 10 ppmv. Future laboratory and airborne intercomparisons are necessary to further understand the remaining differences while helping to identify errors in calibration and measurement techniques.

The NOAA FPH captures monthly water vapor profiles from three sites around the globe. The longest continuous record of upper atmospheric water vapor abundance began in Boulder, Colorado, in 1980. The second site was Lauder, New Zealand, which started monthly soundings in 2004. Hilo, Hawaii, initiated soundings in late 2010. These three sites were chosen to represent the northern and southern mid-latitudes and the tropics, respectively. The typical instrument package flown at these three sites consists of a NOAA FPH, an electrochemical concentration cell (ECC) ozonesonde, and a radiosonde which wirelessly transmits the data back to the receiving station on the ground.

In situ balloon flight profiles complement satellite sensors monitoring the global distribution of water vapor around the planet. Monthly NOAA FPH balloon measurements provide high-vertical-resolution profiles. Satellite sensors, such as the Aura Microwave Limb Sounder (MLS) (Read et al., 2007), measure water vapor in the UTLS and above (Lambert et al., 2007) with almost daily global frequency but with a vertical resolution of 2–3 km. Combining the data from these two measurement techniques provides important distribution information of water vapor for models, to help detect trends, and identify future changes in climate. Differences between the NOAA FPH and the MLS have been explored in depth by Hurst et al. (2014), showing good agreement in the stratosphere (68–26 hPa) at all three NOAA Global Monitoring Division (GMD) sites between August 2004 and December 2012. Previous coincident cryogenic frost point hygrometer (CFH) balloon observations were used in the validation of the earlier versions of the MLS satellite (v1.5 and v2.2) between 2005 and 2007 (Vömel et al., 2007c). Vömel et al. (2007c) found that MLS and CFH profiles agreed to within 2.7 ± 8.7 % from 68 to 22 hPa. The CFH is a small balloon-borne hygrometer developed in 2003 which uses the same fundamental measurement principle as the NOAA FPH (Vömel et al., 2007a). The two instruments have many similarities but also differ in subtle ways including electronics, sunlight filtering, and frost control algorithm.

This paper provides detailed information regarding the description and uncertainty of the current digital FPH along with a brief history of the evolution of the sonde. Comparisons in the laboratory along with dual instrument balloon flights are presented.

## 2 Instrument description and history

**Figure 1:**
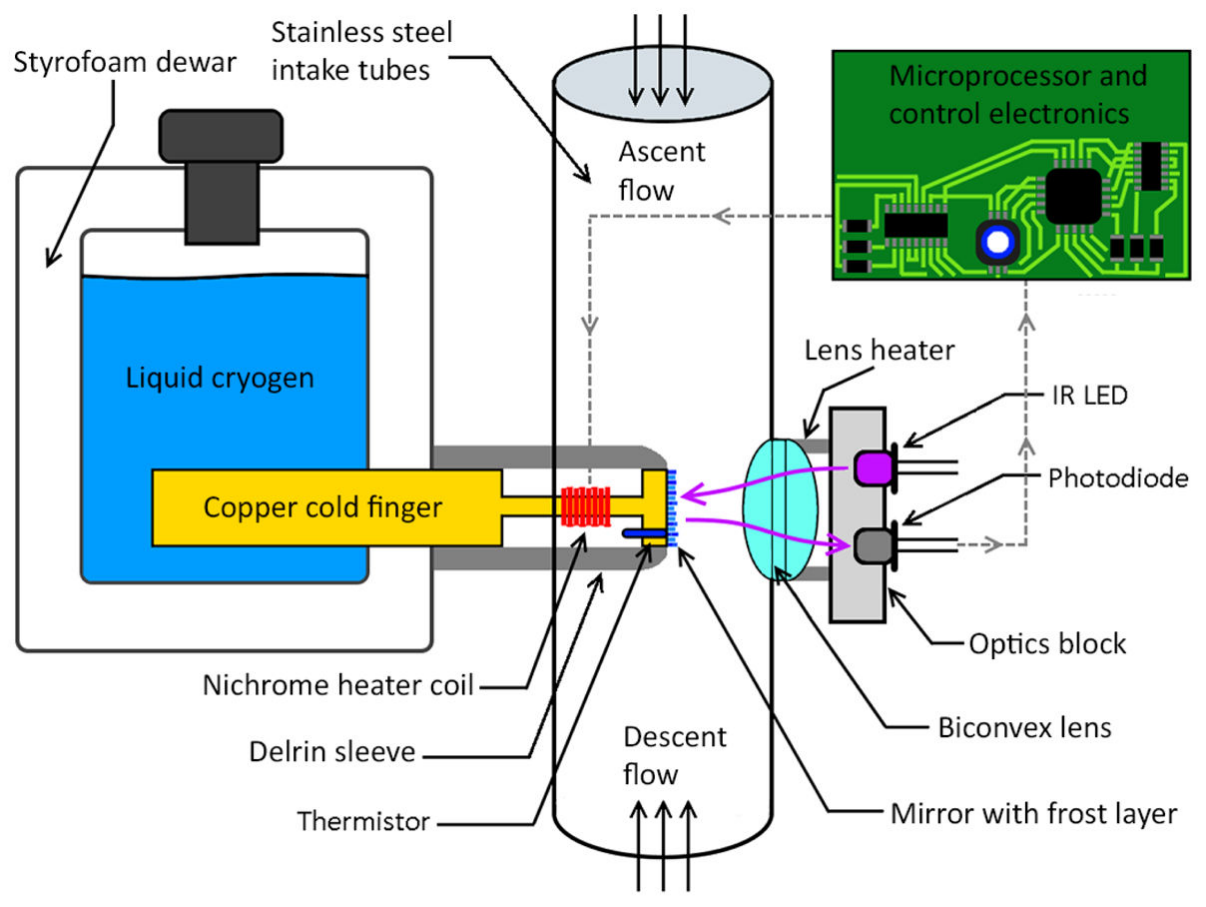
Schematic of the NOAA FPH instrument. The electronics board and optics block are enclosed by insulating foam along with the battery pack while the lens, intake tubes, and mirror head are all exposed to ambient air. The microcontroller uses the photodiode signal to regulate the mirror temperature such that the reflectivity of the frost on the mirror is constant over the flight.

The NOAA FPH is an in situ balloon-borne chilled mirror hygrometer capable of measuring vertical profiles of frost point temperature up to ~ 28 km. The basic measurement principle and calibration method have remained unchanged throughout the time series although significant modernization of the instrument occurred over the ongoing 36 year record (Mastenbrook and Oltmans, 1983; Oltmans, 1985; Vömel et al., 1995; Oltmans and Hofmann, 1995; Hurst et al., 2011a). The chilled mirror principle relies on maintaining a thin, stable layer of condensate on a mirror disk through rapid feedback control. A copper cold finger immersed in a dewar containing liquid cryogen provides cooling power throughout the profile (Fig. 1). A polished mirror disk resides at the opposite end of the cold finger with ambient air passing over it at 3–6 m s^−1^. A nichrome heater wrapped around the narrow shaft of the continuous cold finger and mirror piece provides heat to the mirror. An optical source and detector, composed of an infrared light-emitting diode (LED) and a photodiode, is used to monitor the mirror’s reflectivity as condensate accumulates in the form of dew or frost. A biconvex lens focuses the light returned off the mirror into the photodiode. A calibrated thermistor embedded in the mirror accurately measures the frost point temperature. The frost point temperature is achieved when a stable frost layer is present, showing equilibrium between water vapor in the air sample and the condensate on the mirror. Due to the material and geometry, the surface of the mirror is considered to be uniform in temperature across the 7.1 mm diameter (Mastenbrook and Oltmans, 1983). The Goff–Gratch formulation of the Clausius–Clapeyron equation (Goff and Gratch, 1946; Goff, 1957) is used to calculate the water vapor partial pressure with respect to ice. Dividing the water vapor partial pressure by the dry atmospheric pressure determines the mixing ratio by volume. The chilled mirror principle is unique in that only the thermistor needs to be calibrated against National Institute of Standards and Technology (NIST) traceable temperature standards. This eliminates water vapor calibration scales or standards which are difficult to create, maintain, and use in the field.

The NOAA FPH is flown as a disposable instrument that can be reused if returned. The typical instrument package, including an ozonesonde, radiosonde, and liquid cryogen, is lightweight (< 1.9 kg) and typically flown using a 1200 g latex balloon. Trifluoromethane (CHF3, R23) has been used as the liquid cryogen since the mid-1990s when the switch was made away from CClF3 (R13). Both compounds have ideal physical properties over a wide range of temperatures (−100 to 30 °C) and pressure (10 to 1050 hPa) to provide liquid cooling of the cold finger in all conditions encountered during balloon flights.

The flight train consists of a balloon with a valve in its neck and a parachute attached to a long string unwinder (30–50 m). Nearly all NOAA FPH instrument packages continue to be flown with a valve in the neck of the balloon, allowing slow, controlled descent profiles to be acquired (Mastenbrook, 1966; Kräuchi et al., 2016). The instrument package resides at the bottom of the unwinder that separates the balloon and parachute from the instrumentation in an effort to reduce water vapor contamination from balloon outgassing during the ascent.

Over the 36 years of FPH measurements by NOAA, four versions of the instrument have been flown with each change reflecting advancements in the capabilities of the instrument (Table 1). However, each version adhered to the same NIST-traceable temperature calibration standard.

### 2.1 FPH V1: analog hygrometer

**Figure 2:**
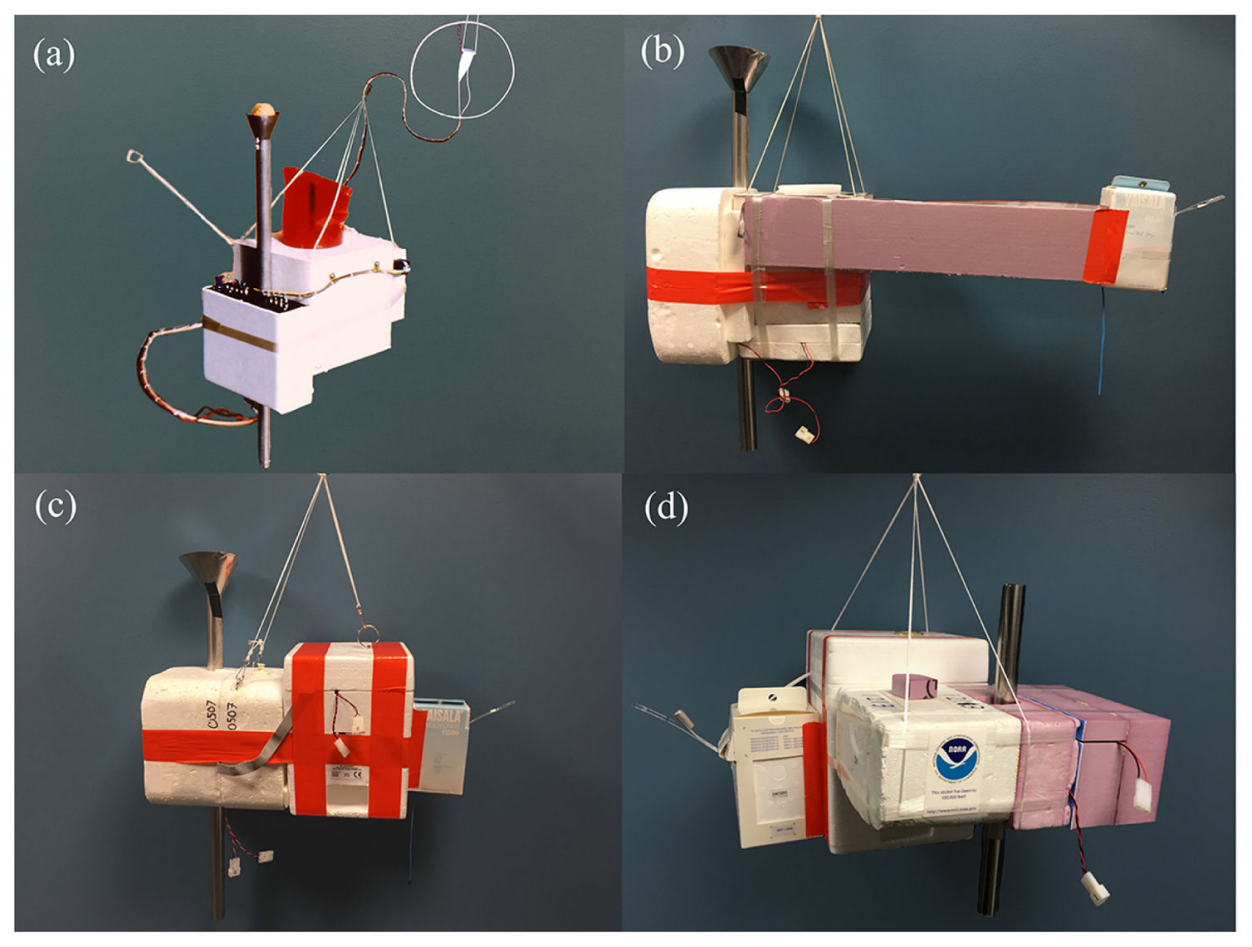
The NOAA FPH V1 hygrometer integrated the baroswitch and electronics from a VIZ “A” radiosonde inside the FPH instrument package with the thermistor protruding on a stick off to the side **(a)**. The VIZ “A” 1680 MHz transmitter (white cone located inside protective loop) was flown 3 m above the instrument to avoid RFI problems. The sun shield for V1 hygrometers utilized a ping pong ball surrounded by a stainless steel shroud to block unwanted sunlight. A 60 cm foam boom separated the Vaisala RS-80 radiosonde and the FPH V2 hygrometer flown without an ECC ozonesonde **(b)**. A Vaisala RS-80 radiosonde and an ECC ozonesonde were attached to the FPH V3 instrument **(c)**. Both V2 and V3 instruments were flown with stainless steel sun shields. The NOAA FPH V4 is currently flown with an InterMet iMet-1-RSB radiosonde and an ECC ozonesonde **(d)**.

Balloon-borne frost point hygrometers have been used to collect vertical profiles of water vapor abundance and distribution in the atmosphere since the late 1950s (Mastenbrook and Dinger, 1961). Monthly profiles were acquired in Washington, DC between 1964 and 1980 using a 4.5 kg balloon-borne frost point hygrometer specifically developed for the program. The water vapor program was moved to Boulder, Colorado, in 1980 following significant instrument improvements that included implementation of solid-state electronics (Mastenbrook and Oltmans, 1983). The components from a 1680 MHz VIZ “A” radiosonde were integrated inside the FPH electronics package, excluding the carbon hygristor humidity sensor, which was intentionally left off (Fig. 2a). This included the specially designed baroswitch and electronics of the radiosonde with the thermistor protruding on a stick off to the side. The baroswitch controlled hygrometer gain changes during the flight and served as the main pressure source for this version of the FPH. The data were telemetered wirelessly and recorded on a paper strip chart. The VIZ “A” radiosonde transmitter was flown 3 m above the rest of the instrument package to avoid radio frequency interference (RFI) (Mastenbrook, 1981).

### 2.2 FPH V2: digital interface to Vaisala RS-80 radiosonde

In February 1991, a digital interface was added utilizing a 403 MHz Vaisala RS-80 radiosonde and a separate 8 bit analog-to-digital converter (ADC) board which was replaced in October 1991 by a 12 bit ADC board. No shifts were detected in the data recorded digitally instead of on the strip chart recorder formerly used with the 1680 MHz VIZ “A” radiosonde (Oltmans et al., 2000). Starting in 1993, several steps were taken to minimize problematic 403 MHz RFI, including adding a 60 cm horizontal foam boom to separate the radiosonde from the other instrumentation and adding ferrite beads to any wires on the FPH (Fig. 2b). Silver mirrors used before 1977 were replaced with rhodium-plated copper mirrors on the Boulder instrument until nickel- and gold-plated copper mirrors became the standard late in 1992. In 1998 the custom-made aneroid barometer incorporated on the FPH became obsolete, forcing its replacement by a solid-state sensor in the new supporting electronics designed in 2000 and improved upon in 2001. This sensor was only used for gain changes as the Vaisala RS-80 radiosonde measured pressure for all calculations for FPH V2 and V3 hygrometers.

### 2.3 FPH V3: lightweight hygrometer with upgraded electronics

In 2004, a modern electronics board was added utilizing surface mount components that eliminated RFI from the attached Vaisala RS-80 radiosonde. Modified mechanical and optical parts were combined with new foam packaging to create an analog FPH boasting a 70 % reduction in weight (Fig. 2c). These improvements enabled the second NOAA/GMD stratospheric water vapor station to be started at Lauder, New Zealand, in 2004.

Prior to the 2004 version of the hygrometer the optical system consisted of two pairs of optical elements in close thermal proximity. The “specular pair” looked down at the mirror through a lens and provided a current proportional to the light reflected off the mirror while the “bias” pair looked directly at each other. The two pairs were matched according to their temperature coefficients and attached to the optics block with thermal epoxy to minimize temperature-driven signal drift in the differential measurement. Before flight, the bias signal was set at 21 % by adjusting a setscrew to match the specular signal setpoint. With the introduction of temperature stabilization of the aluminum optics block the bias pair was deemed unnecessary on all FPH instruments flown after 2004, allowing a fixed resistor to replace the bias pair on the electronics boards.

### 2.4 FPH V4: digital microcontroller hygrometer

Between 2005 and 2008, a digital version of the hygrometer was developed that offered greater precision and ease of use, while decreasing the weight, cost, power consumption, and manufacturing time. The skill required to successfully launch and collect water vapor data with the FPH V4 is equivalent to that needed to prepare and release an ozonesonde with an attached radiosonde, of which there are approximately 3000 flown each year by different research groups around the world. The older analog instrument required more in-depth knowledge and time to successfully produce a high-quality water vapor profile.

A major advancement to the digital FPH was the introduction of the modulating filter that removes unwanted sunlight from the photodiode signal during a daytime balloon flight, similar to the CFH’s earlier implementation (Vömel et al., 2007a). Earlier versions of the FPH utilized a stainless steel sun shield to block the sunlight except for flights done between 1997 and 2003. The sun shield was effective at blocking sunlight but often contributed to stratospheric contamination on the ascent portion of the flight. The new switching sunlight filter provides clean stratospheric frost control by reducing contamination. Since 2010, the FPH LED is modulated at 24 Hz while the photodiode continually measures the signal from the mirror. The microcontroller calculates the difference between the LED on and off signals in real time during the flight. This enables the sunlight captured by the photodiode during the LED off periods to be removed from the LED on signal to maintain unvarying condensate coverage during a flight.

Eliminating the sun shield, incorporating a lens heater, and implementing a flexible frost point-dependent gain schedule (Vömel et al., 2007a) were significant improvements linked with the digital FPH. A digital calibrated pressure sensor was incorporated into the hygrometer, adding the flexibility to perform tasks throughout the flight based on pressure and also served as a backup source when the radiosonde had issues.

Prior to 2008 the LED in the analog version of the FPH had a wide viewing angle and a peak wavelength centered at 940 nm. This LED and the photodiode were replaced with an 880 nm peak LED and a 900 nm centered photodiode, both with narrow viewing angles. Peak wavelength matching is not necessary because the spectral bandwidth at half power is ±40 nm, providing ample overlap. The optics block is temperature controlled to 32.00 ± 0.01 °C throughout the balloon flight. Great care is taken to maintain temperature stability of the photodiode and LED because both are highly temperature sensitive. Since 2009, a lens heater similar to that flown on the CFH (Vömel et al., 2007a) was implemented to help reduce condensation, which may form on the lens in humid conditions.

The FPH is insulated by several pieces of foam, with a 300 mL foam Dewar on one side of the instrument and a foam battery enclosure housing the optics block, electronics board, and battery pack on the opposite side. Very thin, hydrophobic stainless steel inlet tubes with a diameter of 2.25 cm are glued above and below the sensor housing to deliver clean air across the polished mirror disk in the middle of the sensor housing (Fig. 2d).

#### 2.4.1 Mirror feedback controller, electronics, and battery power

The FPH uses a custom-made electronics board with an onboard pressure sensor and an 8 bit Atmel AVR microcontroller. The electronics remain between 20 and 30 °C during a flight by capturing heat from the nearby optics block and batteries. The FPH is powered by a 9 V battery pack with 3.5 Ah capacity. A nichrome heater wrapped around the mirror allows for a maximum heating power of 22 W, more than sufficient to measure dew or frost point temperatures anywhere on Earth when R23 cryogen is used for cooling.

A 16 bit ADC converter, oversampled to 19 bit, provides continuous mirror reflectivity feedback to the microcontroller from the photodiode. A dynamic schedule of proportional–integral–derivative (PID) gain values, based on a slow moving average of the measured frost point temperature, enhances the ability of the FPH to control the frost layer over an extremely wide range (> 10^5^) of water vapor number densities in the atmosphere. The modern gain schedule was developed to allow the instrument to be flown anywhere in the world using the same PID gain table.

The older analog version of the NOAA FPH utilized a simple proportional controller with one gain change within a profile. It was not uncommon for the analog instrument to experience frost control oscillations in the first few kilometers of the profile and directly after the gain change that historically occurred in the middle troposphere. The digital instrument, with the dynamic gain schedule, provides nearly uninterrupted water vapor profile measurements that are used to determine integrated precipitable water columns.

**Figure 3:**
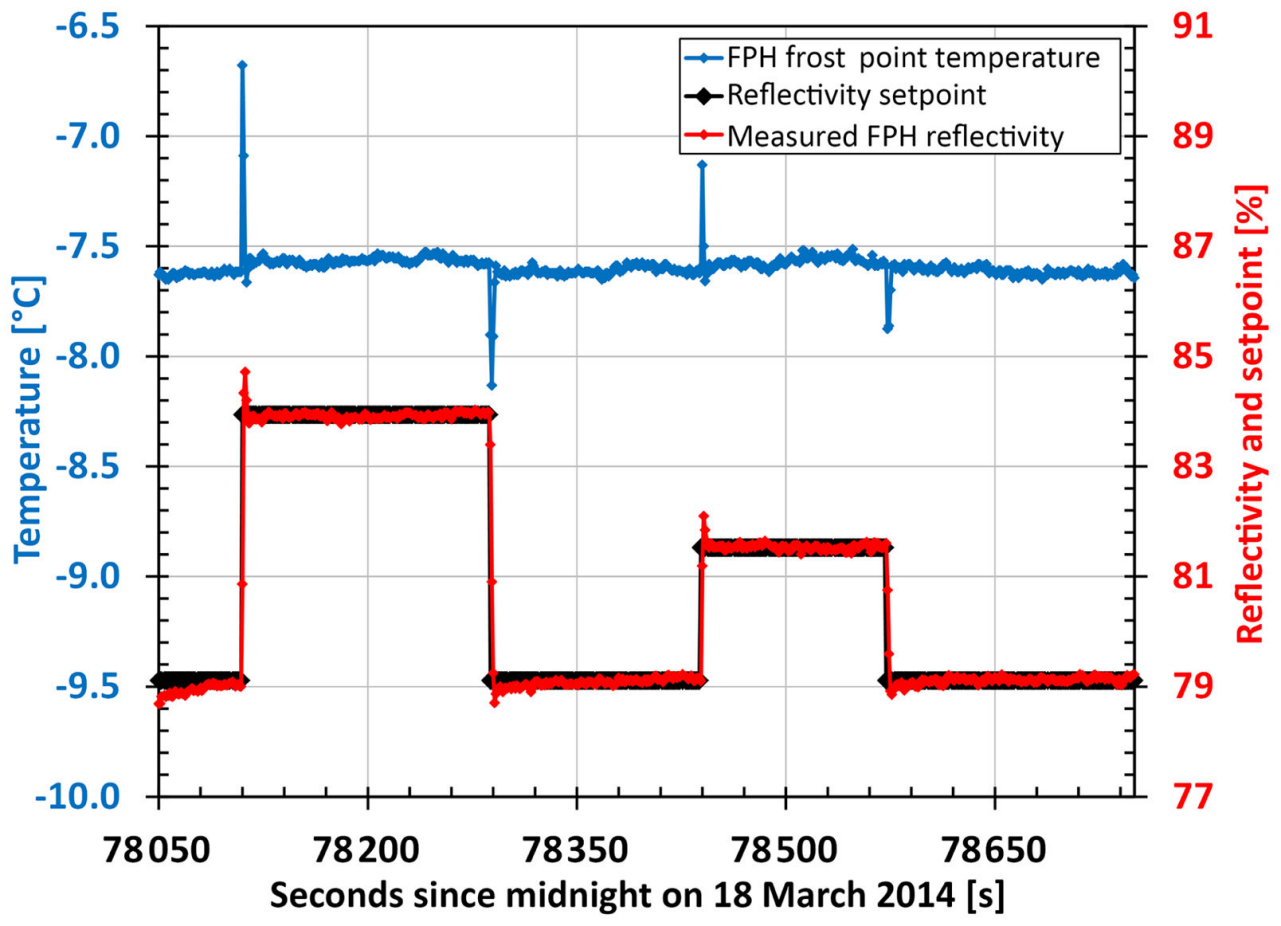
The frost point temperature measured by the NOAA FPH mirror thermistor (blue) remains unchanged when varying the amount of condensate on the mirror by adjusting the reflectivity set-point (black). The measured reflectivity of the mirror is shown in red. Ambient laboratory air was sampled during this experiment.

Under stable conditions the FPH controls dew or ice on the mirror such that 79.1 % of the light that would reflect off a bare mirror reaches the photodiode. This value has remained constant since 1980. Laboratory testing has shown that increasing the setpoint value by > 7 % does not affect the frost point temperature measured by the instrument (Fig. 3). Maintaining a constant condensate layer is much more important than the actual setpoint value used.

#### 2.4.2 Radiosonde telemetry and data collection

The FPH has been flown with the InterMet Systems iMet-1-RSB radiosonde since summer 2009 because the Vaisala RS-80 radiosonde used previously became obsolete. This radiosonde provides measurements of pressure, temperature, and humidity (PTU) (Hurst et al., 2011b) along with any externally connected instrument data. The iMet-1-RSB supplies GPS location information that enables payload tracking as well as the calculation of wind speed and direction.

The iMet-1-RSB transmits 1 Hz wireless data at 1200 baud allowing up to 120 bytes of data to be sent each second. The iMet-1-RSB radiosonde employs an open-source telemetry protocol (XDATA) that allows multiple instruments to be connected via serial daisy chain. Roughly 80 bytes of externally connected instrument data can be sent down along with the PTU and GPS data of the radiosonde. The data are recorded on the ground by a custom-made software program “SkySonde” that was designed and written by NOAA/GMD engineers to receive and demodulate telemetered data from the iMet-1-RSB.

## 3 Thermistor calibration

Although the equipment used to calibrate thermistors has changed over the years, the thermistor calibration method has remained unchanged, providing greater stability in the absolute calibration of FPHs over the decades. Details regarding the different data acquisition systems, curve fitting routines, and NIST-traceable thermometers are discussed below.

The frost point temperature is measured by a bead thermistor embedded in the mirror disk. Each thermistor is individually calibrated in small batches in a 4 L insulated dewar containing pure ethanol. A laboratory stirrer uniformly maintains the alcohol bath temperature during the calibration. The bath temperature is measured with a NIST-traceable thermometer. The thermistors are inserted approximately 16 cm into the alcohol bath and are attached to a digital multimeter outside of the dewar. Between 1980 and 2013, finely ground dry ice chips were manually added to the bath to cool and maintain the alcohol temperature at three targets: 0, −45, and −79 °C. Great care was taken while adding small amounts of dry ice to maintain a ±0.005 °C tolerance around each of the three setpoints to minimize curve fitting errors. Calibration coefficients were calculated from the measured resistances at the three target temperatures and used in an equation to calculate mirror temperature during a flight (Clark, 1961). Starting in 2014, calibrations were done by continuously measuring the bath temperature and the resistance of each thermistor as the alcohol bath slowly warmed from −93 to +19 °C. The new continuous resistance measurement technique utilizes a six-point fit instead of the historic three-point fit eliminating the extrapolation curve fitting correction necessary for the older three-point fit (see Sect. 3.1 below).

A NIST-traceable Hewlett-Packard quartz thermometer was employed as the temperature reference between 1980 and 1998. This probe had accuracies of ±0.075 °C between −80 and −50 °C and ±0.040 °C between −50 and +150 °C with a resolution of 0.001 °C over the full range. When the quartz thermometer broke in 1998 a platinum resistance thermometer (PRT, Hart Scientific, USA) became the temperature reference for all calibrations after 1998. This probe has a stated accuracy of ±0.01 °C with 0.001 °C resolution over the full range of NOAA FPH thermistor calibrations. The Hart PRT probe has been recalibrated by the manufacturer every 6 years since 1998 and still serves as the NIST-traceable standard today.

Until late 1998 the resistance of each thermistor was hand recorded from an HP digital multimeter. Then a new data acquisition system was developed using a Campbell Scientific CR10 data logger that allowed 20 thermistors to be calibrated at once. During the summer of 2004 an Agilent 34970A data acquisition switching unit was set up as the new primary calibration system, allowing 40 thermistors to be calibrated at a time.

### 3.1 Three-point thermistor extrapolation correction below −79 °C frost point temperature: 1980–2013

**Figure 4:**
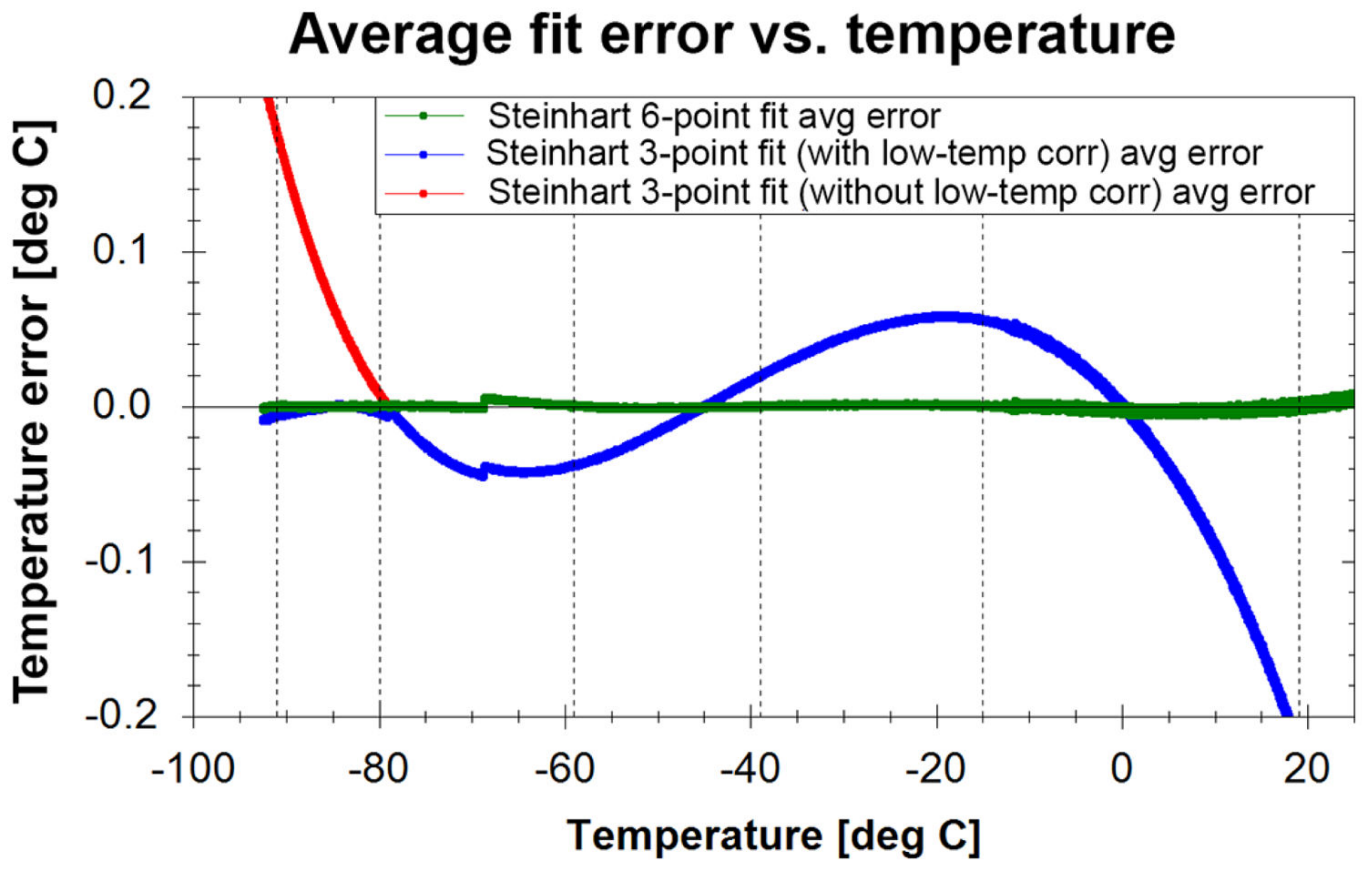
Difference between two different calibration fits and the actual measured bath temperature during a calibration run. Each curve is comprised of the mean of 39 thermistors fit errors. The Steinhart–Hart three-point fit is shown with (blue) and without (red) the low temperature correction. All flights between 1980 and 2013 utilize the three-point fit with the low temperature correction. Flights starting in 2014 do not need a low temperature correction since the new six-point thermistor fit (green) was implemented. Dashed lines show the six calibration points.

The traditional three-point thermistor curve fit was utilized for thermistor calibrations performed from 1980 to 2013. Between 0 and −79 °C the curve fit was better than ±0.06 °C (Fig. 4) that translates to at most a 0.5–1.1 % difference in water vapor mixing ratio over the entire flight (+20 to −93 °C) or in terms of absolute differences 0.06 ppmv when analyzing stratospheric data. Colder than −79 °C there existed a one-sided extrapolation error with a maximum error of 0.16 °C at −90 °C equivalent to an absolute difference of 0.16 ppmv or < 3 % in water vapor mixing ratio. Based on a batch of six thermistors calibrated down to −93.3 °C, a linear correction Eq. (1) was applied to frost point temperatures colder than −79 °C for all NOAA FPH flights prior to 1991 (Mastenbrook, 1981):

(1) $$T_{\text{FpT},\text{corr}}=1.013(T_{\text{FpT}})+1.046,$$

where *T*_FpT_ is in °C. Scherer et al. (2008) developed an improved empirical fit to correct frost point temperatures below −79 °C for all flights between 1991 and the last three-point thermistor calibration in 2013. The correction was based on the 30 thermistor calibration from −100 to 20 °C (Vömel et al., 2007b).

(2) $$T_{\text{FpT},\text{corr}}=T_{\text{FpT}}-{\left(-0.029(T_{\text{FpT}}+79)+0.083\right)}^{2}$$

Both corrections slightly lowered frost point temperatures < −79 °C and reduced water vapor mixing ratios < 3 % in the stratosphere. After the Scherer et al. (2008) correction was applied the calibration curve fits between 1991 and 2013 were better than ±0.06 °C over the full temperature range.

No corrections were applied to the frost point temperatures > 0 °C for any of the FPH data over this period as they would be much smaller than the uncertainties of the FPH measurements in the lower troposphere.

### 3.2 Thermistor self-heating correction applied to FPH instruments calibrated before 1987

Prior to 1987, all thermistor calibrations were performed using an HP3490A multimeter that applied a known current to each thermistor and determined its resistance by measuring the voltage drop. For resistances < 10 kΩ, the meter applies an excitation current of 0.85 mA that is large enough to heat the small FPH thermistors by ~ 1.5 °C at the 0 °C calibration point. Calibrations performed after 1987 used a different multimeter with a 0.1 mA excitation current, reducing the self-heating. The self-heating at the two colder calibration points (−45 and −79 °C) was minimal because both old and new multimeters applied smaller excitation currents at those calibration points.

The self-heating problem at the 0 °C setpoint was documented during a calibration in 1987. A group of six thermistors was calibrated using the original 0.85 mA excitation current and the lower 0.1 mA setting. When using the three-point calibration fit the warm bias at 0 °C produces a cold bias of < 0.21 °C at frost point temperatures of −90 °C (Scherer et al., 2008). Utilizing these data, Scherer et al. (2008) derived an empirical correction that was applied to FPH frost point temperatures < −79 °C for all flights before 1987:

(3) $$T_{\text{FpT},\text{corr}}=T_{\text{FpT}}+{\left(-0.0203(T_{\text{FpT}}+61.9)\right)}^{2}-0.119.$$

Applying the empirical correction increases water vapor mixing ratios by < 4 % at −90 °C. All data published since 2008 have these two corrections applied where applicable.

### 3.3 Six-point thermistor calibration: 2014 to present

In 2014 a new calibration technique was implemented. Instead of manually maintaining the calibration bath at 0, −45, and −79 °C, the bath is initially cooled to −93 °C with liquid nitrogen and allowed to slowly warm (Vömel et al., 2007a), eliminating the need to extrapolate below −79 °C. An expanded version of the Steinhart–Hart formulation now derives fifth-order polynomial fits of thermistor resistances to mirror temperature (Steinhart and Hart, 1968) at six calibration temperatures: −91, −80, −59, −39, −15, and −19 °C. These temperatures were selected to minimize fit errors, with greater weighting for the important stratospheric temperature range. The NIST-traceable thermometer temperatures and the resistance values for all 40 channels are recorded continuously on an Agilent 34970A digital multimeter until the bath reached > 19 °C, a process that takes ~ 50 h because the de-war is well insulated. The probe temperature is measured before and after the two second sweep (< 0.003 °C temperature change during each sweep) of each thermistor and then interpolated to determine the probe temperature during each of the 40 thermistor measurements. The new six-point fit reduces errors over the full range to better than ±0.01 °C compared to ±0.06 °C for the historical three-point fit (Fig. 4). The new six-point fit converts to at most a 0.08–0.2 % difference in water vapor mixing ratio over the entire flight or 0.01 ppmv, in terms of absolute differences, when looking specifically in the stratosphere. The discontinuity near −70 °C is caused by the custom data acquisition software switching between the 1 MΩ and the 100 kΩ range setting on the Agilent 34970A digital multimeter in order to measure the lower thermistor resistances as the bath warms. The discontinuity does not affect the balloon flight data as there are no calibration points near −70 °C and the mirror thermistor is measured with the onboard ADC, which does not have this issue.

Though far more comprehensive, the new continuous-measurement calibration technique has a complication due to the changing temperature of the bath as it warms. The PRT probe has a larger thermal mass than the thermistors and therefore warms at a slower rate. At each sampling time, the measured probe temperature will be slightly colder than the true temperature of the thermistors. To account for this thermal “lag” a small correction is required.

**Figure 5:**
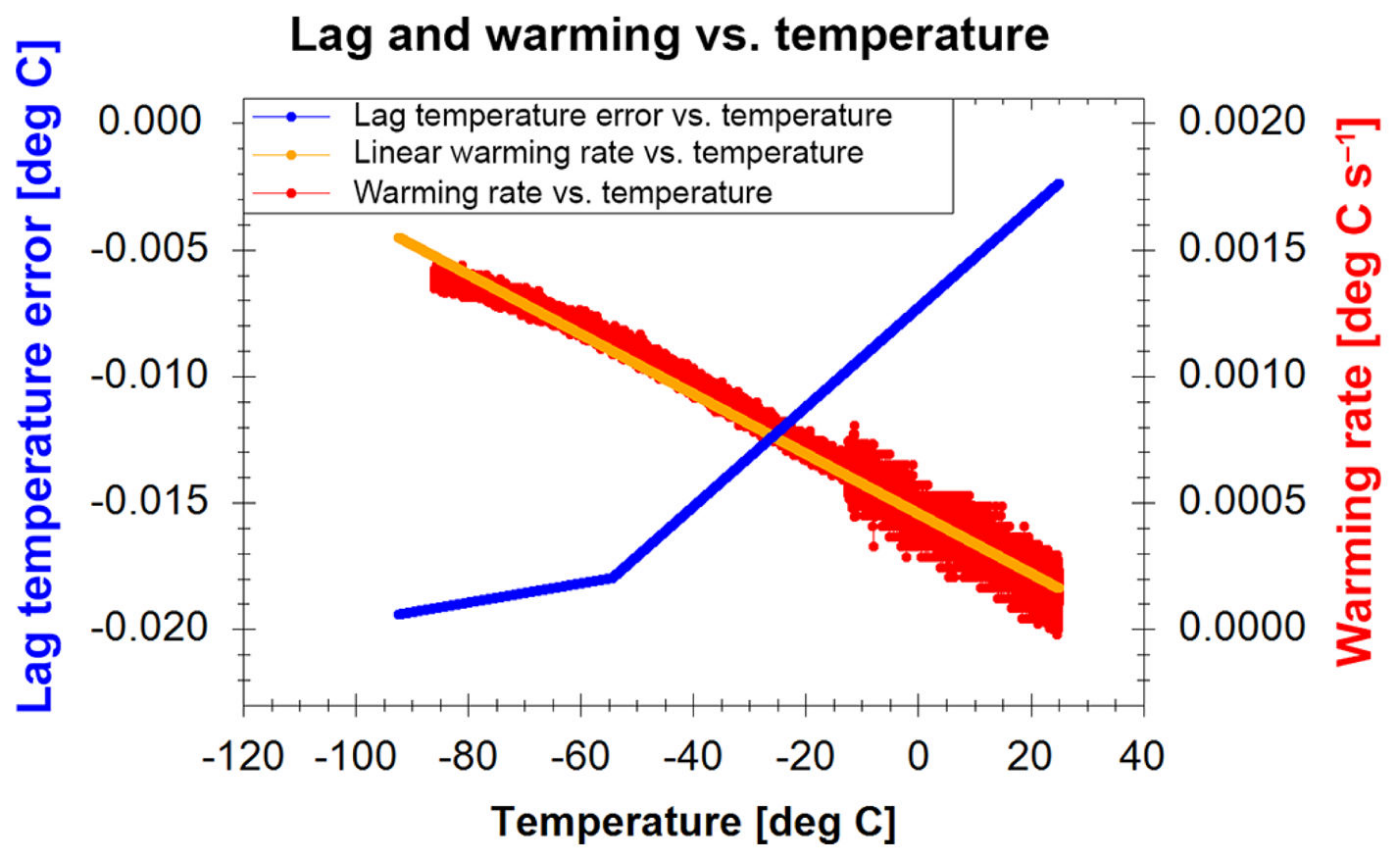
Temperature correction applied to raw NIST-traceable PRT thermometer readings to account for the lag between the larger PRT thermometer and the small thermistors in the alcohol bath.

The thermal lag correction was determined by performing both a historical three-point calibration and a new continuous-measurement calibration on the same set of thermistors. At the three historical static temperature setpoints there is no thermal lag of the PRT probe despite its larger thermal mass by holding the bath in equilibrium for an extended time at each temperature (> 3 min). The results are compared to those from the new continuous-measurement calibration to determine the thermal lag correction for the probe. The correction is dependent on the warming rate of the bath, so an equation is created mapping warming rate to temperature lag correction for use in future calibrations (which may warm at different rates due to varying lab conditions and equipment). Before applying the lag correction, a line is fit to the calculated warming rate to avoid inserting extra noise into the corrected probe temperature data (Fig. 5).

## 4 Measurement uncertainty

Errors and uncertainties are treated as outlined in the “Guide to the expression of uncertainty in measurement” by the working group 1 of the Joint Committee for Guides in Metrology (JCGM/WG 1, 2008). Uncertainty in the FPH measurements is determined through the evaluation of random and systematic errors. Uncontrolled factors in measurements that cause normally distributed fluctuations are characterized as random errors and their uncertainties can be reduced by increasing the number of measurements. An example of a random uncertainty in the FPH is the frost control stability. Alternatively, systematic errors remain regardless of the number of measurements, producing biases in the measurements if not corrected. Even if systematic errors are properly understood and corrected there will be a residual error as no correction is perfect. The uncertainty of the mirror temperature measurement by the FPH electronics board is an example of a systematic error. All of the FPH uncertainties are summarized in Table 2.

The largest measurement uncertainties during a sounding arise from instabilities in frost control. The magnitudes of these terms are calculated using the standard error of the estimate of frost point temperature over a fixed interval (0.25 km) to determine the residual error from the linear fit (2 *σ*). The uncertainty is calculated for the balloon ascent and the descent separately. The uncertainty over the 0.25 km interval range is between 0.1 to 1.5 °C in the profile, depending on the instrument performance at any given time.

The mirror temperature uniformity and location of the thermistor embedded in the mirror have been demonstrated to induce systematic uncertainties < 0.1 °C in the CFH frost point temperatures (Vömel et al., 2007a). Uncertainties for the FPH are the same because it uses the same thermistors as the CFH and has mirrors of the same thickness and diameter.

The uncertainty of the mirror temperature measurement on the FPH circuit board varies with the frost point temperature due to the ADC. These uncertainties, along with the mirror temperature uniformity and location uncertainties, are combined in quadrature to produce the manufacturing uncertainty in each instrument.

Thermistor calibration fit, calibration repeatability, and the reference thermometer uncertainty are combined in quadrature to yield total uncertainty estimates for both the three-point and six-point calibration fits. All FPHs since 2014 use the six-point fit and have a combined calibration uncertainty of 0.03 °C. All prior instruments using the three-point fit have a combined uncertainty of 0.1 °C before 1990 and 0.07 °C after 1990 and before the six-point fit.

**Figure 6:**
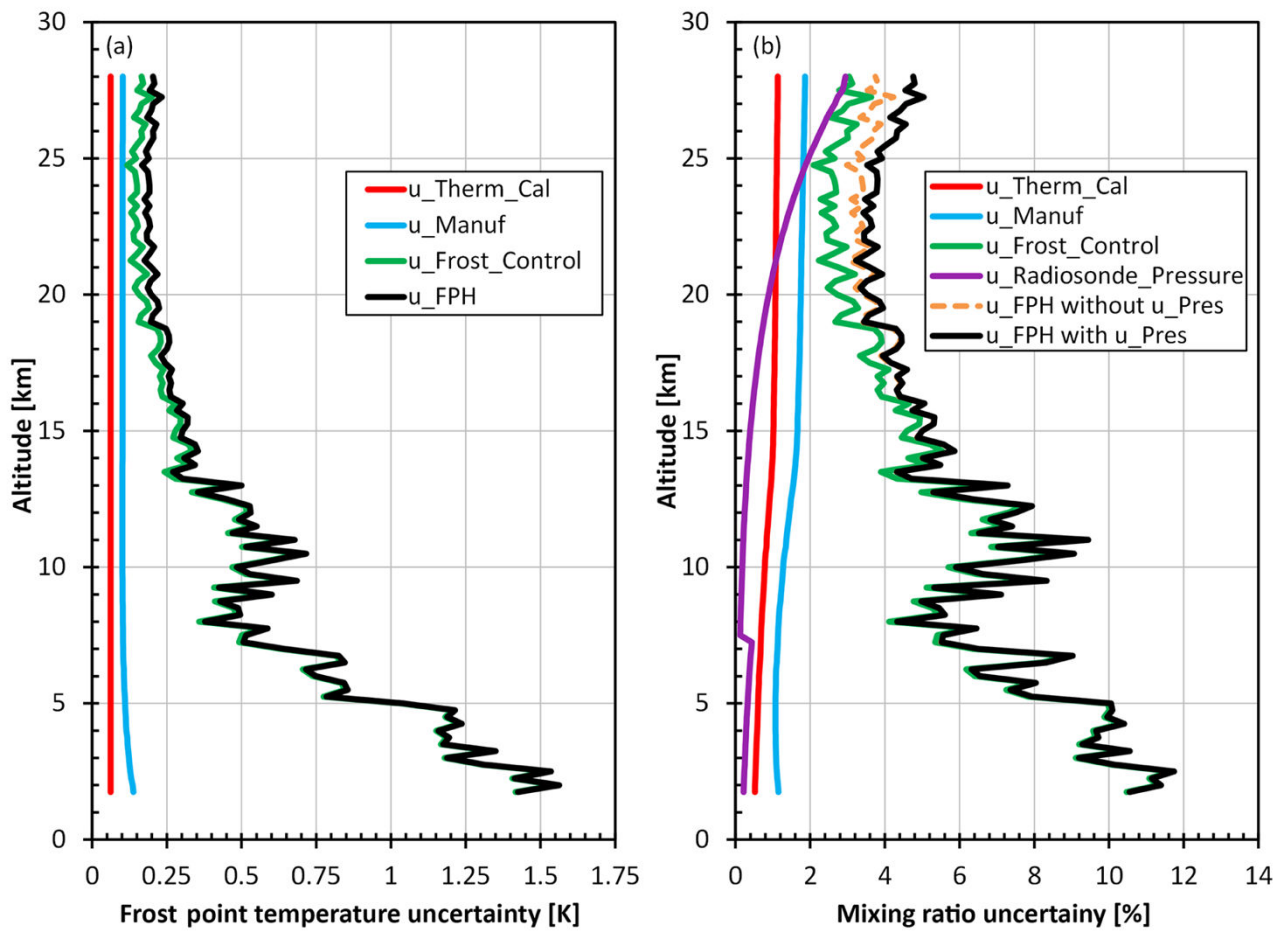
Contributions of specific uncertainty terms to the total FPH uncertainties in frost point temperature **(a)** and water vapor mixing ratio **(b)**. The FPH uncertainties shown are the mean values from 24 flights in Boulder, CO. Radiosonde pressure uncertainties (magenta) are based on InterMet’s sensor specifications that decrease at 8 km, producing the discontinuity.

Two years of FPH data from Boulder, Colorado, are analyzed to show the contributions of the various uncertainty estimates in Fig. 6. Panel a shows the average in frost point temperature (K) with frost control stability uncertainties dominating the total to 15 km with the other two terms contributing more above 15 km to the top of the flight. The mixing ratio uncertainties shown in Fig. 6b include those computed from the manufacturer’s specifications for radiosonde pressure sensor uncertainties in magenta, and the total with and without the pressure sensor uncertainty (black and dashed gray lines, respectively). Manufacturer-quoted radiosonde pressure sensor uncertainties are used for the iMet-1-RSB (1070–400 hPa: ±1.8 hPa; 400–4 hPa: ±0.5 hPa), the Vaisala RS-80 (1080–3 hPa: ±1.0 hPa), and the VIZ “A” (1050–5 hPa: ±2.0 hPa) at the 2 *σ* accuracy limit (Stauffer et al., 2014; Richner and Phillips, 1981; Tarasick et al., 2016). The uncertainties below 15 km are < 12 % due to both atmospheric variability and less stable frost control. In the stratosphere total uncertainties are < 6 % (Fig. 6b).

## 5 Mirror condensate

Condensate, either dew or ice, deposits on the mirror when cryogen is added before the flight. Even at mirror temperatures 0 to −40 °C, supercooled liquid water may reside on the mirror before homogeneous nucleation will spontaneously freeze the liquid. The uncertain phase of the condensate between 0 and −40 °C is inherent to all chilled mirror frost point hygrometers (Fujiwara et al., 2003; Vömel et al., 2007a). Correct identification of the condensate phase is important because the vapor pressures above liquid and ice are very different within this temperature range.

**Figure 7:**
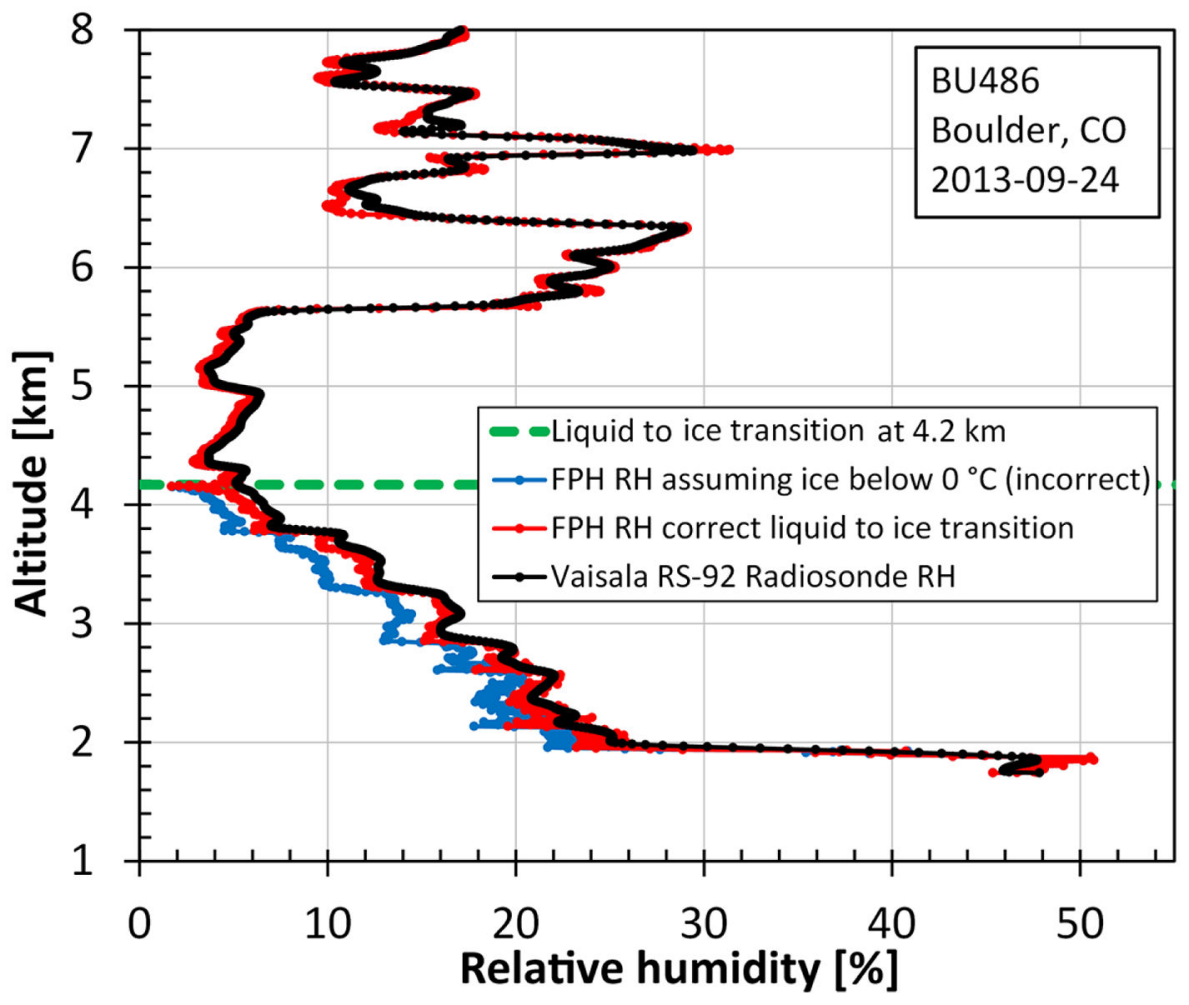
The liquid to ice transition on the FPH mirror can be seen at 4.2 km on the ascent. Using the appropriate vapor pressure formulations below and above the point of condensate transition brings the RH values of the FPH and the Vaisala RS-92 into agreement.

The NOAA FPH compares the relative humidity measured by the attached radiosonde to help determine the phase of the condensate on the mirror. Radiosondes measure atmospheric relative humidity with a thin film polymer capacitive sensing surface. This sensor always reports relative humidity with respect to liquid and does not have ambiguity issues like chilled mirror frost point hygrometers. Relative humidity profiles are calculated from the FPH mirror temperatures (0 to −40 °C) using the different vapor pressure equations for liquid and ice (Fig. 7), then each is compared to the radiosonde’s relative humidity profile to identify when the liquid to ice transition occurs on the mirror. When the condensate starts as liquid this transition occurs around −31 °C. The transition is not instantaneous and may exhibit a lag of as much as 2 min during some flights. A sharp dip in the frost point temperature occurs when the condensate changes to ice, further helping to distinguish the correct transition point.

According to Murphy and Koop (2005) metastable forms of ice, such as cubic and amorphous ice, may exist colder than 200 K in the atmosphere. These have a higher vapor pressure than the most stable form, hexagonal ice. At temperatures > 200 K only hexagonal ice is formed on the frost point hygrometer mirror. Below the 200 K threshold cubic ice nucleates first; this is estimated to have a vapor pressure 3 to 11 % greater than hexagonal ice, depending on which enthalpy measurement is used. The cubic ice eventually transforms to hexagonal ice over a period of minutes to days. If the ice on the mirror is cubic instead of hexagonal the frost point temperatures must bias warm to compensate for the greater equilibrium vapor pressure above cubic ice. This leads to high biases in the partial pressures calculated from the Goff–Gratch equation and therefore high biases in the water vapor mixing ratios. For this reason, during every balloon ascent when the frost point temperature reaches −53 °C, the ice on the mirror is completely sublimed by a heat pulse (mirror clear) followed by rapid frost regrowth within 50 s, as is done during each CFH profile (Vömel et al., 2007a). Hexagonal ice is quickly reformed on the mirror and is maintained for the duration of the flight because transformation into metastable ice is not possible under atmospheric conditions. The mirror clear is performed at −53 °C (220 K), providing a large window to safely grow hexagonal ice instead of cubic ice which can be formed below 200 K. This procedure helps to eliminate ambiguity during a flight. Prior to 2008 the older instruments switched from low to high gain around −55 °C, creating a similar sublimation and regrowth of the frost layer due to frost control oscillations instead of the dedicated mirror clear at −53 °C. It is unlikely that cubic ice or any other metastable form of ice forms on the FPH mirror after the mirror clear or gain change. To date, there have not been any profiles where cubic ice has been identified.

Thornberry et al. (2011) conducted experiments using a laboratory version of the CFH to study the potential interference of gas-phase nitric acid (HNO_3_) on chilled mirror frost point hygrometers. Mixtures of moist air with up to 4 ppbv of HNO_3_ for 150 min presented no detectable change in the measured mirror temperatures, showing it is unlikely that HNO_3_ can change the frost point temperatures significantly during a typical atmospheric sounding.

## 6 Pressure activated balloon valve system

Obtaining uncontaminated water vapor profiles during a slow descent (i.e., no balloon burst) is accomplished using a valve situated in the neck of a balloon. This technology has been utilized on frost point hygrometer flights since 1964 when Mastenbrook (1966) developed a reliable and simple valve system to release helium from the balloon. A fitting on the valve in the neck of an expandable latex balloon was opened at a predetermined pressure. The valve system evolved out of necessity for two main reasons. First, moisture captured on the balloon during ascent in the wet troposphere can contaminate the ascending hygrometer measurements. This contamination is from outgassing of the balloon and flight train in the dry stratosphere and is intermittent because the FPH pendulums in and out of the wake of the balloon. Once the valve opens the balloon ascent slows then starts to descend, and now the FPH leads the flight train with clean uncontaminated air below. Equally important, the valve provides slow and constant descent rates of ~ 5 m s^−1^ compared to a burst balloon that descends rapidly (> 40 m s^−1^) in the stratosphere despite the parachute. The parachute is attached in case the valve fails to open or the balloon bursts before the designated pressure has been attained.

Kräuchi et al. (2016) show an example of stratospheric contamination on an ascent profile starting near 25 km with uncontaminated measurements from the descent extending the profile above 27 km. When analyzing 141 flights from Lauder, New Zealand, instruments flown without a sun shield encounter contamination starting in the stratosphere below 25 km ~ 15 % of the time whereas instruments flown with a sun shield prior to 2010 see contamination ~ 52 % of the time. Although eliminating the sun shield has significantly improved stratospheric data collection with regards to contamination on the ascent, the controlled uncontaminated valved descent profiles continue to provide stratospheric data reaching above the contaminated ascent data.

The current version of the valve system consists of two pieces: a PVC valve and a digital microcontroller circuit board with a calibrated pressure sensor (Kräuchi et al., 2016). The 175 g valve is constructed from PVC pipe with a plastic lid secured with waxed string. Separate battery packs are used to power the electronics board and to cut the waxed string with a heated nichrome wire at the desired pressure. Custom software allows the user to set the cut down pressure and apply a one-point pressure offset determined from a laboratory pressure standard. The overall accuracy of the pressure sensor, after the one-point offset is applied, is ±1 hPa with 0.01 hPa resolution. For profiles over Boulder, Lauder, and Hilo the valves are set to open at 16.0 hPa, or roughly 28.5 km.

The new version of the valve and electronics has increased the success rate from 76 to 94 % for all balloon flights between 1991 and 2014 that did not burst before the cut down pressure was achieved. At the same time the new system decreased the valve system weight by 60 %, which saves helium.

## 7 Intercomparisons 7.1 AquaVIT-2

The Karlsruhe Institute of Technology (KIT) operates a large volume (84 m^3^) aerosol and cloud simulation chamber (AIDA) with the ability to vary pressure, temperature, and water vapor mixing ratio in a highly controlled fashion. In April 2013 a 2-week-long water vapor measurement inter-comparison experiment (AquaVIT-2) was performed at the AIDA chamber. AquaVIT-2 built off a previous intercomparison (AquaVIT-1; Fahey et al., 2014) at the AIDA chamber in October 2007 in which the NOAA FPH did not participate. A special laboratory version of the balloon FPH was constructed specifically for AquaVIT-2 to externally measure chamber air extracted through a heated tube by a downstream pump. The laboratory instrument has three main differences from the balloon version. First, liquid nitrogen was used as the cryogen in the laboratory instrument, requiring a Teflon sleeve to partially insulate the copper cold finger from the colder liquid nitrogen. Next, the sensor housing and intake tubing were modified to connect to the heated extraction tube without any leaks since most of the chamber experiments were done at sub-ambient pressures. Finally, the flow rates through the laboratory instrument were reduced when compared to the balloon sonde to conserve the chamber air.

**Figure 8:**
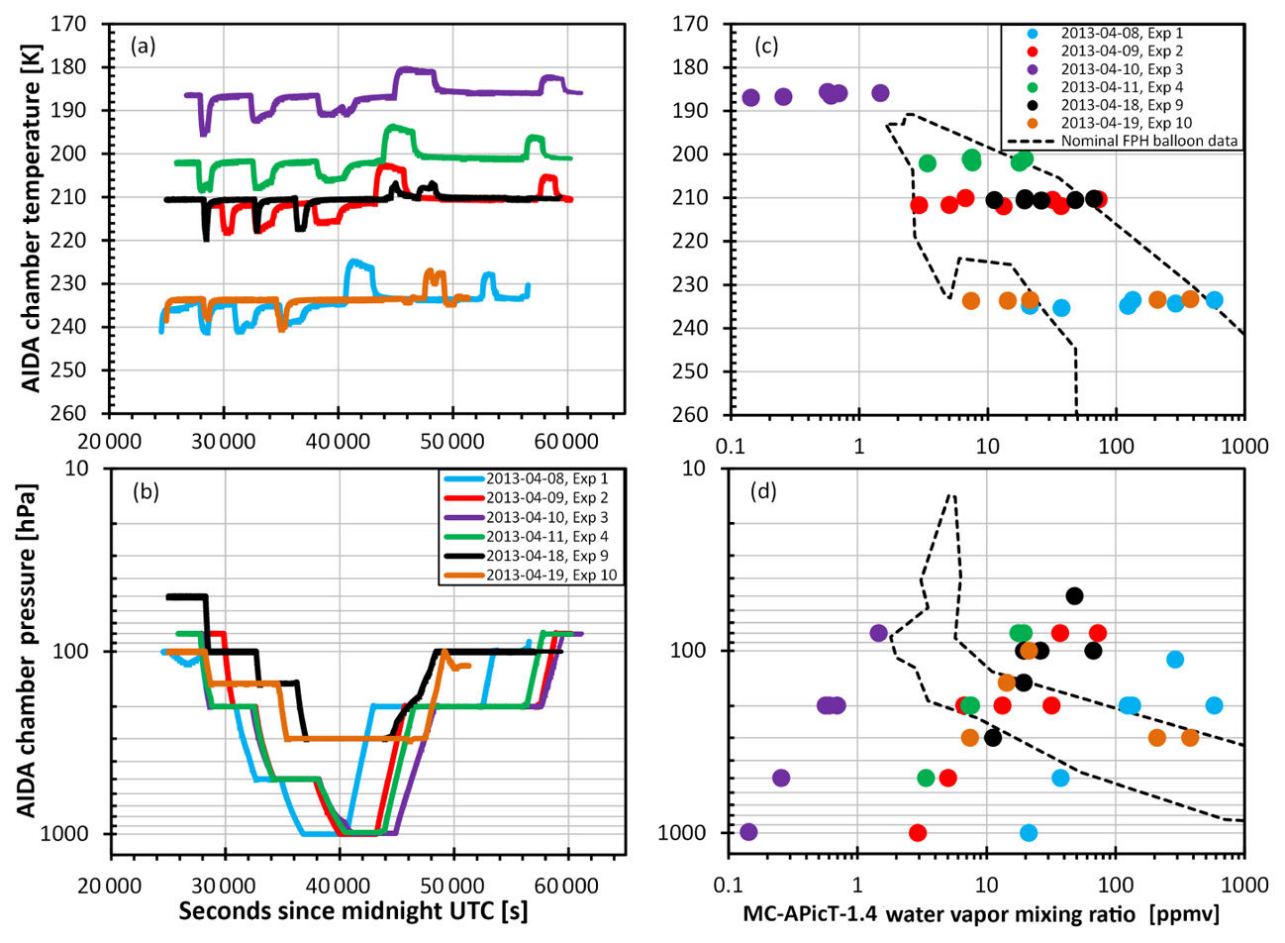
Daily time series of temperatures **(a)** and pressures **(b)** for all data during the six non-blind experiments are shown. The relationship between chamber temperature and pressure with 36 individual stable water vapor mixing ratio segments is shown in panels **(c)** and **(d)**, respectively. Areas contained by dashed lines indicate the range of water vapor and temperature in the actual atmosphere.

The purpose of AquaVIT-2 was to intercompare state-of-the-art and prototype atmospheric hygrometers with each other and with independent humidity standards to determine precision and accuracy of the different instruments. Each day the chamber was held at one specific temperature while pressure and water vapor mixing ratio were changed throughout the day. Daily chamber temperatures ranged from 190 to 233 K over the 10-day experiment (Fig. 8a). Dynamic temperature excursions of nearly ±10 K, caused by adiabatic expansion and compression when air was removed from or added to the chamber, were not included in the analysis. The water vapor mixing ratios varied between ~ 0.15 and 1000 ppmv while the pressure in the chamber started each day between 50 and 100 hPa and increased stepwise to a maximum of 300–1000 hPa before stepping back down to 80–100 hPa at the end of each day. Typically there were six distinct pressure steps throughout the day to intercompare the instruments during quasi-static conditions. Temperature and pressure in the chamber were stable within ±0.3 K (1 *σ*) and ±0.35 hPa (1 *σ*), respectively, during each of the 36 measurement segments examined. Three of the 10 experiment days were established as blind intercomparison days when data were sent directly to independent and impartial referees. Here we focus on comparing 6 of the 7 non-blind days between the NOAA FPH and the MC-APicT-1.4, an open-path, in-cloud, direct tunable diode laser absorption spectrometer (dTDLAS) (Kühnreich et al., 2015). MC-APicT-1.4 is a successor of the initial APICT instrument described in Ebert et al. (2005) and selectively measures, without any gas sampling process, the interstitial water vapor mixing ratio within the AIDA chamber (even within an ice cloud) with help from an open-path white cell integrated into the AIDA chamber. All MC-APicT-1.4 data were evaluated by the German National Metrology Institute (PTB) based on a proprietary, calibration-free first-principle dTDLAS approach. The validity of this data evaluation was recently successfully demonstrated by comparing PTB’s extractive, airborne dTDLAS field hygrometer, SEALDH, with the German primary humidity standard at PTB (Buchholz et al., 2014). The 3 blind days are excluded because the data are still not available.

**Figure 9:**
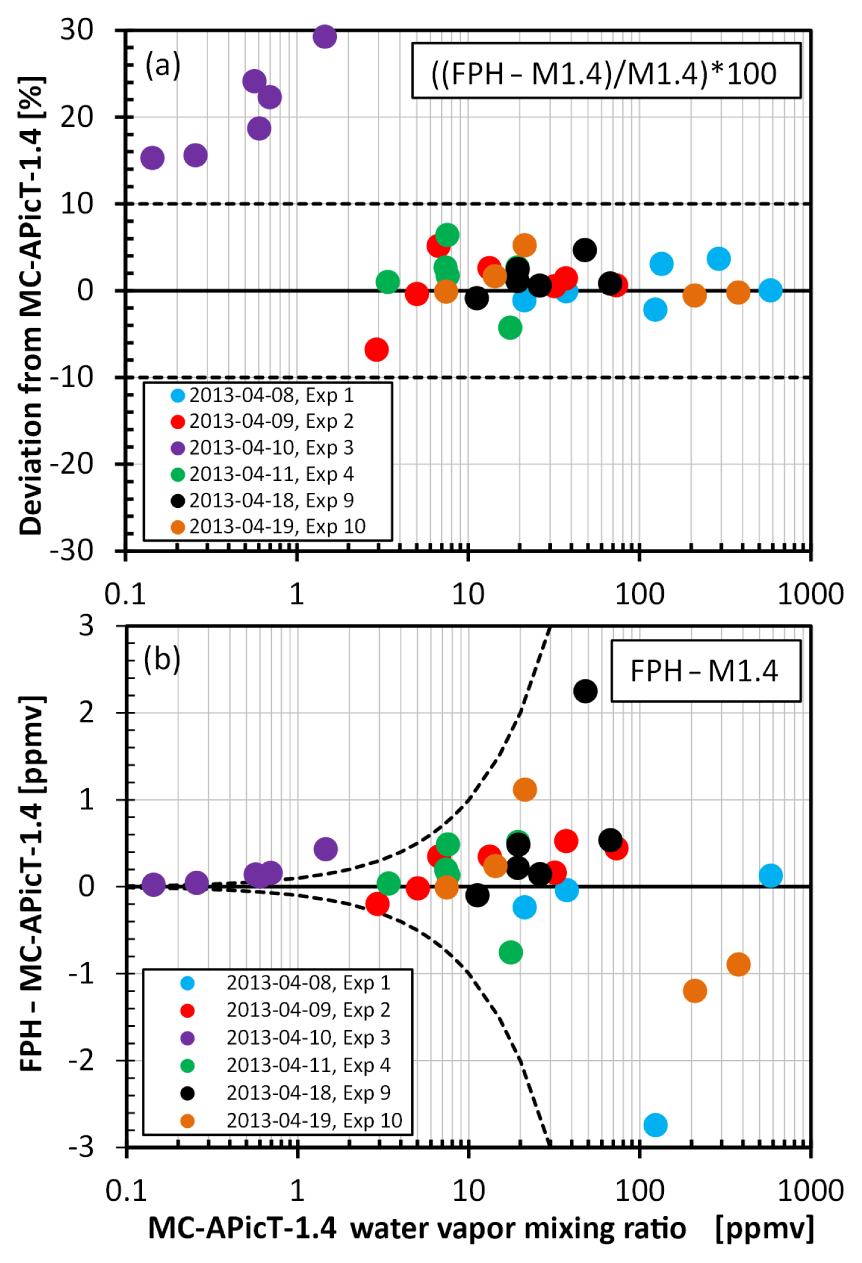
Difference between NOAA FPH and MC-APicT-1.4 mixing ratios in **(a)** relative % and **(b)** absolute (ppmv) units for mixing ratios between 0.1 and 600 ppmv over the 6 days of non-blind experiments.

Temperatures and water vapor mixing ratios during nearly all the static air temperature segments were within the observed range measured by the NOAA FPH in the atmosphere. The exception was experiment 3 on 10 April 2013 (Fig. 8c), which was colder and drier than the real stratosphere. Only a quarter of the water vapor mixing ratios during stable pressure segments were within the range observable by the NOAA FPH (Fig. 8d). A total of 36 stable sections were analyzed, each lasting an average of 28 min. Excluding data from experiment 3, the agreement between the FPH and the MC-APicT-1.4 instrument was 1.0 ± 5.5 % (2 *σ*) for realistic atmospheric conditions between 2 and 600 ppmv. From here forward, the uncertainties presented with mean values are always twice the standard deviation. Below 1.5 ppmv the agreement is better than 30 % (Fig. 9a). Although the 15–30 % differences for experiment 3 appear to be large, the absolute differences are small, ranging from 0.02 to 0.43 ppmv (Fig. 9b).

**Figure 10:**
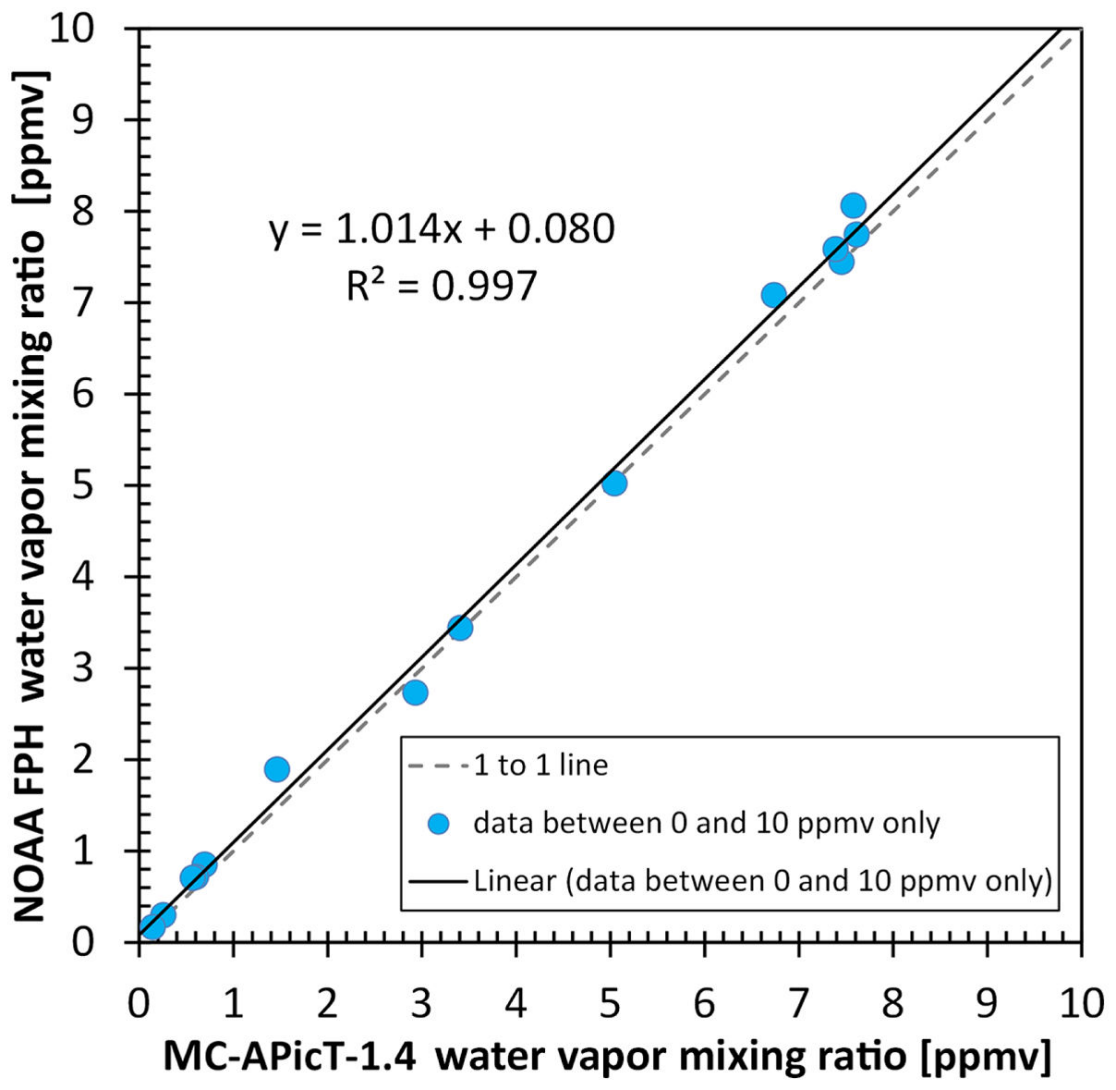
Correlation plot showing all data collected between 0.1 and 10 ppmv over the 6 static non-blind days of experiments. A linear trend line (black) is shown with slope and offset.

The FPH data collected over the non-blind days in the region of interest (0.1–10 ppmv) show good agreement with the MC-APicT-1.4 instrument. Even including the six stable sections < 1.5 ppmv where the FPH was measuring 15–30 % higher than MC-APicT-1.4, the linear trend between the two instruments shows a slope of 1.014 and near-zero *y* intercept with minimal scatter as seen in Fig. 10.

### 7.2 Dual frost point hygrometer balloon flights

**Figure 11:**
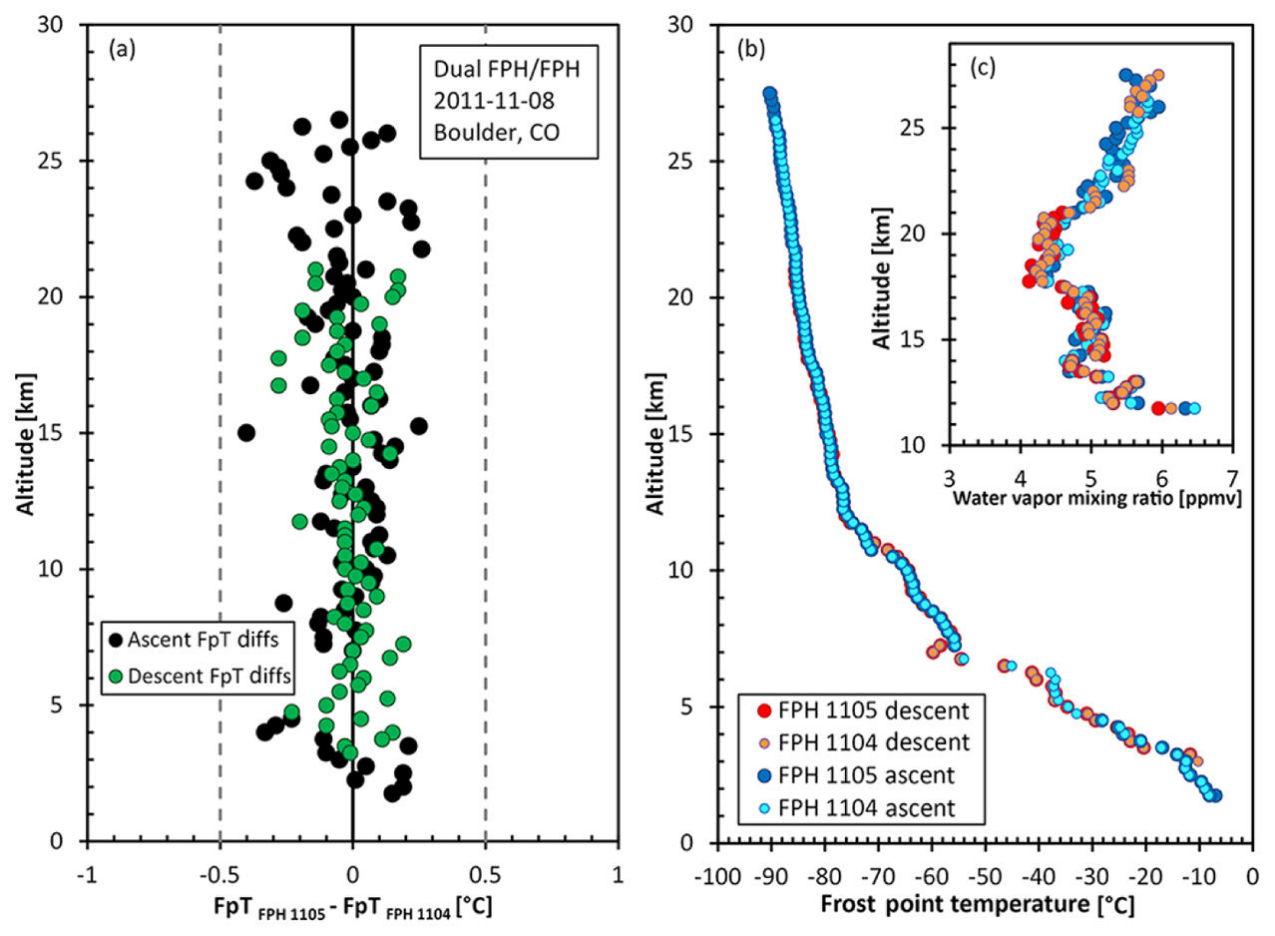
Frost-point temperature differences from a dual FPH flight on the same balloon in Boulder, CO, are shown for the ascent (black) and descent (green). The frost point profiles **(b)** along with the stratospheric water vapor mixing ratios between 3 and 7 ppmv **(c)** show agreement over the entire flight.

To exclude the atmospheric variability and pressure measurement differences, two FPHs were flown using a single balloon and radiosonde on 8 November 2011. When comparing ascent and descent data the mean differences in frost point temperature and water vapor mixing ratio (0.25 km bin averages) between the two hygrometers were −0.019 ± 0.25 °C and −0.16±3.9 %, respectively (Fig. 11). Instrument “1105” had frost control oscillations after the valved balloon turn on this flight, resulting in missing descent data until 21 km. Although the descent data often capture high-quality data above the ascent data, there are times when the descent data suffer, depending on atmospheric conditions or unknown instrumental issues. In these cases, the descent data are flagged bad and removed during quality control after the flight.

**Figure 12:**
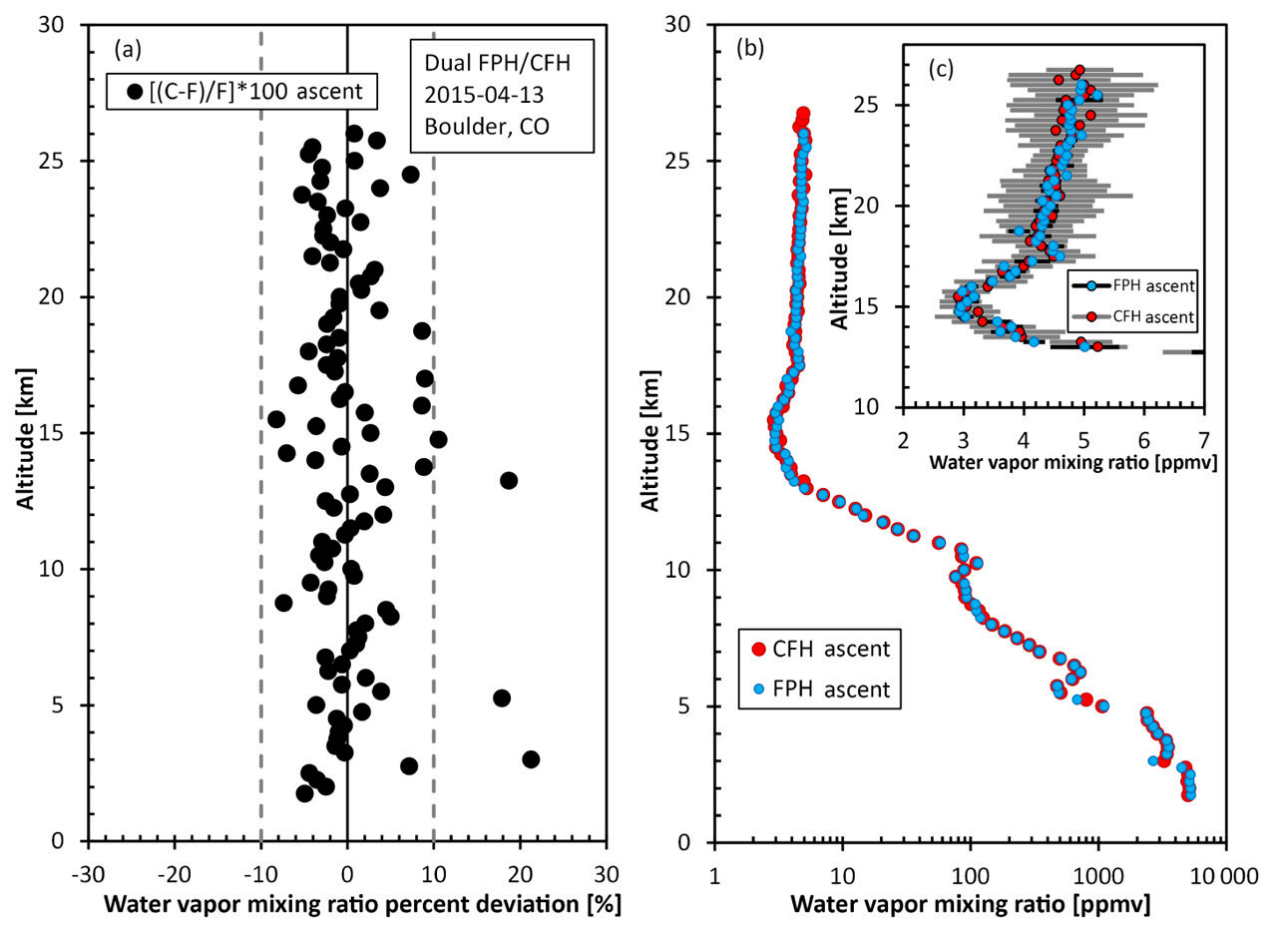
Mixing ratio deviations are shown between the CFH and FPH **(a)** beside the full stratospheric water vapor mixing ratio plot **(b)**. Stratospheric water vapor mixing ratios between 2 and 7 ppmv are show in panel **(c)** where the error bars are ±1 *σ* for the 0.25 km bins in gray (CFH) and black (FPH).

Similarly, a dual flight with a CFH and an FPH using a single radiosonde and balloon was flown in Boulder, Colorado, on 13 April 2015 (Fig. 12). The agreement in water vapor mixing ratio was 0.35 ± 10 % even when taking into account the differences larger than 10 % that occurred when the mixing ratio was changing quickly. Only ascent data were compared due to an unexplained cryogen loss at the top of the flight. The CFH is tuned to be a fast responding instrument with some oscillations in order to quickly respond to changes in water vapor during a profile. The error bars are larger on the CFH compared to the FPH during this flight (Fig. 12c) partially due to incomplete sunlight filtering coupled with fast responding stratospheric PID controller values (Vömel et al., 2016).

Figure 11a and b are plotted in absolute frost point temperature and frost point temperature differences to show the principal raw measurement of the instrument over an entire profile. However, the CFH FPH dual flight data are plotted in mixing ratio and mixing ratio percent difference to show the full range of water vapor mixing ratio measured during a flight.

## 8 Summary

Since 1980 the NOAA FPH has acquired high-quality monthly vertical profiles of water vapor over Boulder, CO. The instrument has evolved over the years but the physical measurement principal and the calibration process has not deviated, making the stability of FPH measurement accuracy suitable for long-term monitoring and process studies. In 2004, a second FPH sounding site was added at Lauder, New Zealand, initiating what is now the longest upper-atmospheric water vapor record in the Southern Hemisphere. A third site at Hilo, Hawaii, was started late in 2010 to measure tropical water vapor profiles, complementing the measurement programs at the existing northern and southern midlatitude locations.

Improvements to the microcontroller version of the instrument in 2008 lead to more high-quality flights at the three NOAA/GMD water vapor sites. The biggest improvements to the FPH were implementing digital sunlight filtering to eliminate the sun shield, adding a lens heater, and creating a frost point-dependent gain schedule.

The various contributions to the uncertainty of the instrument are calculated for each profile. The overall uncertainty in the stratosphere is typically < 6 %, increasing to < 12 % in the troposphere. The majority of the uncertainty estimate resides in the frost control stability during a flight.

In 2014 a new continuous mirror thermistor calibration technique was adopted. This allows a six-point calibration fit that reduces fit errors to ±0.01 °C compared to ±0.06 °C for the older three-point method. A lag correction was also developed to account for the larger PRT thermometer, with a slower response time than the tiny FPH thermistors. This correction was determined by comparing the results from the old and new calibration techniques for the same batch of 39 thermistors.

Comparison data during the AquaVIT-2 chamber experiment in Karlsruhe, Germany, with the MC-APicT-1.4 direct tunable diode laser spectrometer show good agreement over 6 non-blind days of experiments. For water vapor mixing ratios between 2 and 600 ppmv the agreement was 1.0 ± 5.5 %. Dual FPH/FPH and FPH/CFH balloon flights show excellent consistency between instruments and do not give any indication of a bias.

## 9 Data availability

Vertical water vapor profiles for Boulder, Lauder, and Hilo are available on the GMD public ftp: ftp://aftp.cmdl.noaa.gov/data/ozwv/WaterVapor/. AquaVIT-2 data are not yet available for the public. Per the data agreement signed by the campaign participants, all data sets will be released to the public at a date to be agreed on by the referees and the instrument principal investigators. No date has been determined at this time.

## Figures and Tables

**Table 1:** The four versions of the NOAA FPH flown between the present day and 1980 along with the radiosondes, weights, and data acquisition method.

FPH version	Period	Radiosonde frequency and model	Data acquisition method	Weight without cryogen (g)
FPH V1	1980–1991	1680 MHz VIZ “A”	Analog strip chart recorder	1550
FPH V2	1991–2004	403 MHz Vaisala RS-80	Digital Strato software	1500
FPH V3	2004–2008	403 MHz Vaisala RS-80	Digital Strato software	475
FPH V4	2008–present	403 MHz InterMet iMet-1-RSB	Digital SkySonde Client/Server	450

**Table 2:** Overview of the sources contributing to the FPH uncertainty budget. Combining the frost stability, manufacturing variability, and thermistor calibration uncertainties in quadrature can be calculated for a fixed interval in the FPH profile.

Uncertainty parameter	Value	R/S^1^
Frost control stability, u_Frost_Control	0.1–1.5 K	R
Temperature uniformity of the mirror, u_Mirror_ Uniform	< 0.1 K	S
FPH electronics board thermistor measurement, u_FPH_ ADC	< 0.02 K (stratosphere), < 0.17 K (troposphere)	S^2^
Manufacturing uncertainty total, u_Manuf	= (u_Mirror_Uniform^2^+ u_FPH_ADC^2^)^1/2^	S^2^
Thermistor calibration fit, u_Cal_Fit	0.06 K (three-point fit), 0.01 K(six-point fit)	S
Thermistor calibration repeatability, u_Cal_Repeat	0.043 K (three-point fit), 0.028 K (six-point fit)	S
Reference thermometer, u_Ref_Therm	0.01 K after 1998, 0.075 K before 1998	S
Thermistor calibration total, u_Therm_Cal	= (u_ Cal_Fit^2^+ u_Cal_Repeat^2^+ u_Ref_ Therm^2^)^1/2^, 0.1 K < 1990, 0.07 K > 1990 (three-point fit), 0.03 K (six-point fit)	S^3^
NOAA FPH uncertainty total, u_FPH	= (u_ Frost_ Control^2^+ u_Manuf^2^+ u_Therm_Cal^2^)^1/2^	

1 Random or systematic;

2 dependent on frost point temperature;

3 constant in profile.

## References

[R1] Brewer AW (1949). Evidence for a world circulation provided by the measurements of helium and water vapor distribution in the stratosphere, Q. J Roy Meteor Soc.

[R2] Buchholz B, Böse N, Ebert V (2014). Absolute validation of a diode laser hygrometer via intercomparison with the German national primary water vapor standard. Appl Phys B.

[R3] Clark LE (1961). Report of the determination of exactness of fit of thermistor to the equations logR = A + B/(T + 2) and logR = A + B/(T + 2) + CT. Tech Rep 2168.

[R4] Ebert V, Teichert H, Giesemann C, Saathoff H, Schurath U (2005). Fibre-Coupled In-situ Laser Absorption Spectrometer for the Selective Detection of Water Vapour Traces down to the ppb-Level. Tm – Technisches Messen.

[R5] Dessler AE, Zhang Z, Yang P (2008). Water-vapor climate feedback inferred from climate fluctuations, 2003–2008. Geophys Res Lett.

[R6] Fahey DW, Gao R-S, Möhler O, Saathoff H, Schiller C, Ebert V, Krämer M, Peter T, Amarouche N, Avallone LM, Bauer R, Bozóki Z, Christensen LE, Davis SM, Durry G, Dyroff C, Herman RL, Hunsmann S, Khaykin SM, Mackrodt P, Meyer J, Smith JB, Spelten N, Troy RF, Vömel H, Wagner S, Wienhold FG (2014). The AquaVIT-1 intercomparison of atmospheric water vapor measurement techniques. Atmos Meas Tech.

[R7] Forster PM, Shine KP (2002). Assessing the climate impact of trends in stratospheric water vapor. Geophys Res Lett.

[R8] Fujiwara M, Shiotani M, Hasebe F, Vömel H, Oltmans SJ, Ruppert PW, Horinouchi T, Tsuda T (2003). Performance of the Meteolabor “SnowWhite” chilled mirror hygrometer in the tropical troposphere: Comparisons with the Vaisala RS-80 A/H humicap sensors. J Atmos Ocean Tech.

[R9] Goff JA (1957). Saturation pressure of water on the new Kelvin temperature scale, T. Am Soc Heating Ventilating Eng.

[R10] Goff JA, Gratch S (1946). Low-pressure properties of water from −160 to 212 F, T. Am Soc Heating Ventilating Eng.

[R11] Held IM, Soden BJ (2000). Water vapour feedback and global warming, Annu. Rev Energ Env.

[R12] Hurst DF, Oltmans SJ, Vömel H, Rosenlof KH, Davis SM, Ray EA, Hall EG, Jordan AF (2011a). Stratospheric water vapor trends over Boulder, Colorado: Analysis of the 30 year Boulder record. J Geophys Res.

[R13] Hurst DF, Hall EG, Jordan AF, Miloshevich LM, Whiteman DN, Leblanc T, Walsh D, Vömel H, Oltmans SJ (2011b). Comparisons of temperature, pressure and humidity measurements by balloon-borne radiosondes and frost point hygrometers during MOHAVE-2009. Atmos Meas Tech.

[R14] Hurst DF, Lambert A, Read WG, Davis SM, Rosenlof KH, Hall EG, Jordan AF, Oltmans SJ (2014). Validation of Aura Microwave Limb Sounder stratospheric water vapor measurement by the NOAA frost point hygrometer. J Geophys Res-Atmos.

[R15] (2008). JCGM/WG 1: Evaluation of measurement data – Guide to the expression of uncertainty in measurement.

[R16] Kley D, Russell JM, Philips C (2000). SPARC Assessment of Upper Tropospheric and Stratospheric Water Vapour.

[R17] Kräuchi A, Philipona R, Romanens G, Hurst DF, Hall EG, Jordan AF (2016). Controlled weather balloon ascents and descents for atmospheric research and climate monitoring. Atmos Meas Tech.

[R18] Kühnreich B, Wagner S, Habig JC, Möhler O, Saathoff H, Ebert V (2015). Time-multiplexed open-path TDLAS spectrometer for dynamic, sampling-free, interstitial H_2_ 18O and H_2_ 16O vapor detection in ice clouds. Appl Phys B.

[R19] Lambert A, Read WG, Livesey NJ, Santee ML, Manney GL, Froidevaux L, Wu DL, Schwartz MJ, Pumphrey HC, Jimenez C, Nedoluha GE, Cofield RE, Cuddy DT, Daffer WH, Drouin BJ, Fuller RA, Jarnot RF, Knosp BW, Pickett HM, Perun VS, Snyder WV, Stek PC, Thurstans RP, Wagner PA, Waters JW, Jucks KW, Toon GC, Stachnik RA, Bernath PF, Boone CD, Walker KA, Urban J, Murtagh D, Elkins JW, Atlas E (2007). Validation of the Aura Microwave Limb Sounder middle atmosphere water vapor and nitrous oxide measurements. J Geophys Res.

[R20] Lee J, Yang P, Dessler AE, Gao B-C, Platnick S (2009). Distribution and radiative forcing of tropical thin cirrus clouds. J Atmos Sci.

[R21] Mastenbrook HJ (1966). A control system for ascent-descent soundings of the atmosphere. J Appl Meteorol.

[R22] Mastenbrook HJ (1981). Operation Manual for Model 1012 Hygrometer.

[R23] Mastenbrook HJ, Dinger JE (1961). Distribution of Water Vapor in the Stratosphere. J Geophys Res.

[R24] Mastenbrook HJ, Oltmans SJ (1983). Stratospheric water vapor variability for Washington, DC/Boulder, CO: 1964–82. J Atmos Sci.

[R25] Meyer J, Rolf C, Schiller C, Rohs S, Spelten N, Afchine A, Zöger M, Sitnikov N, Thornberry TD, Rollins AW, Bozóki Z, Tátrai D, Ebert V, Kühnreich B, Mackrodt P, Möhler O, Saathoff H, Rosenlof KH, Krämer M (2015). Two decades of water vapor measurements with the FISH fluorescence hygrometer: a review. Atmos Chem Phys.

[R26] Mote PW, Rosenlof KH, McIntyre ME, Carr ES, Gille JC, Holton JR, Kinnersley JS, Pumphrey HC, Russell JM, Waters JW (1996). An atmospheric tape recorder: The imprint of tropical tropopause temperatures on stratospheric water vapor. J Geophys Res.

[R27] Müller R, Kunz A, Hurst DF, Rolf C, Krämer M, Riese M (2016). The need for accurate long-term measurements of water vapor in the upper troposphere and lower stratosphere with global coverage. Earth’s Future.

[R28] Murphy DM, Koop T (2005). Review of the vapour pressures of ice and supercooled water for atmospheric applications, Q. J Roy Meteor Soc.

[R29] Oltmans SJ (1985). Measurements of water vapor in the stratosphere with a frost-point hygrometer. Moisture and Humidity-1985-Measurement and Control in Science and Industry.

[R30] Oltmans SJ, Hofmann DJ (1995). Increase in lower-stratospheric water vapour at a mid-latitude northern hemisphere site from 1981 to 1994. Nature.

[R31] Oltmans SJ, Vömel H, Hofmann DJ, Rosenlof KH, Kley D (2000). The increase in stratospheric water vapor from balloonborne, frostpoint hygrometer measurements at Washington, D.C., and Boulder, Colorado. Geophys Res Lett.

[R32] Randel WJ, Wu F, Vömel H, Nedoluha GE, Forster Forster P (2006). Decreases in stratospheric water vapor after 2001: Links to changes in the tropical tropopause and the Brewer-Dobson circulation. J Geophys Res.

[R33] Read WG, Lambert A, Bacmeister J, Cofield RE, Christensen LE, Cuddy DT, Daffer WH, Drouin BJ, Fetzer E, Froidevaux L, Fuller R, Herman R, Jarnot RF, Jiang JH, Jiang YB, Kelly K, Knosp BW, Kovalenko LJ, Livesey NJ, Liu H-C, Manney GL, Pickett HM, Pumphrey HC, Rosenlof KH, Sabounchi X, Santee ML, Schwartz MJ, Snyder WV, Stek PC, Su H, Takacs LL, Thurstans RP, Vömel H, Wagner PA, Waters JW, Webster CR, Weinstock EM, Wu DL (2007). Aura Microwave Limb Sounder upper tropospheric and lower stratospheric H_2_O and relative humidity with respect to ice validation. J Geophys Res.

[R34] Richner H, Phillips PD (1981). Reproducibility of VIZ radiosonde data and some sources of error. J Appl Meteorol.

[R35] Rohs S, Schiller C, Riese M, Engel A, Schmidt U, Wetter T, Levin I, Nakazawa T, Aoki S (2006). Long-term changes of methane and hydrogen in the stratosphere in the period 1978–2003 and their impact on the abundance of stratospheric water vapor. J Geophys Res.

[R36] Rollins AW, Thornberry TD, Gao RS, Smith JB, Sayres DS, Sargent MR, Schiller C, Krämer M, Spelten N, Hurst DF, Jordan AF, Hall EG, Vömel H, Diskin GS, Podolske JR, Christensen LE, Rosenlof KH, Jensen EJ, Fahey DW (2014). Evaluation of UT/LS hygrometer accuracy by intercomparison during the NASA MACPEX mission. J Geophys Res-Atmos.

[R37] Rosenlof KH, Reid GC (2008). Trends in the temperature and water vapor content of the tropical lower stratosphere: Sea surface connection. J Geophys Res.

[R38] Scherer M, Vömel H, Fueglistaler S, Oltmans SJ, Staehelin J (2008). Trends and variability of midlatitude stratospheric water vapour deduced from the re-evaluated Boulder balloon series and HALOE. Atmos Chem Phys.

[R39] Solomon S, Rosenlof KH, Portmann RW, Daniel JS, Davis SM, Sanford TJ, Plattner G-K (2010). Contributions of stratospheric water vapor to decadal changes in the rate of global warming. Science.

[R40] Stauffer RM, Morris GA, Thompson AM, Joseph E, Coetzee GJR, Nalli NR (2014). Propagation of radiosonde pressure sensor errors to ozonesonde measurements. Atmos Meas Tech.

[R41] Steinhart IS, Hart R (1968). Calibration curves for thermistors. Deep-Sea Res.

[R42] Tarasick DW, Davies J, Smit HGJ, Oltmans SJ (2016). A re-evaluated Canadian ozonesonde record: measurements of the vertical distribution of ozone over Canada from 1966 to 2013. Atmos Meas Tech.

[R43] Thornberry T, Gierczak T, Gao RS, Vömel H, Watts LA, Burkholder JB, Fahey DW (2011). Laboratory evaluation of the effect of nitric acid uptake on frost point hygrometer performance. Atmos Meas Tech.

[R44] Vömel H, Oltmans SJ, Hofmann DJ, Deshler T, Rosen JM (1995). The evolution of the dehydration in the Antarctic stratospheric vortex. J Geophys Res.

[R45] Vömel H, David DE, Smith K (2007a). Accuracy of tropospheric and stratospheric water vapor measurements by the cryogenic frost point hygrometer: Instrumental details and observations. J Geophys Res.

[R46] Vömel H, Yushkov V, Khaykin S, Korshunov L, Kyro E, Kivi R (2007b). Intercomparison of stratospheric water vapor sensors: FLASH-b and NOAA/CMDL frost point hygrometer. J Atmos Ocean Tech.

[R47] Vömel H, Barnes JE, Forno RN, Fujiwara M, Hasebe F, Iwasaki S, Kivi R, Komala N, Kyrö E, Leblanc T, Morel B, Ogino S-Y, Read WG, Ryan SC, Saraspriya S, Selkirk H, Shiotani M, Valverde Canossa J, Whiteman DN (2007c). Validation of Aura Microwave Limb Sounder water vapor by balloon-borne Cryogenic Frost point Hygrometer measurements. J Geophys Res.

[R48] Vömel H, Naebert T, Dirksen R, Sommer M (2016). An update on the uncertainties of water vapor measurements using cryogenic frost point hygrometers. Atmos Meas Tech.

[R49] Weinstock EM, Smith JB, Sayres DS, Pittman JV, Spackman JR, Hintsa EJ, Hanisco TF, Moyer EJ, StClair JM, Sargent MR, Anderson JG (2009). Validation of the Harvard Lyman- in situ water vapor instrument: Implications for the mechanisms that control stratospheric water vapor. J Geophys Res.

